# Robust and Interpretable Metagenomic Modeling Through Structure-Aware Multi-View Learning and Attribution-Guided Biological Insight

**DOI:** 10.21203/rs.3.rs-9956795/v1

**Published:** 2026-08-05

**Authors:** Lindong Jiang, Martha Isabel Gonzalez-Ramirez, Kuan-Jui Su, Xiao Zhang, Anqi Liu, Chuan Qiu, Zhe Luo, Qing Tian, Lei Huang, Chaoyang Zhang, Hui Shen, Hong-Wen Deng

**Affiliations:** 1 Tulane Center of Biomedical Informatics and Genomics, School of Medicine, Tulane University, New Orleans, LA, 70112, United States; 2 School of Computing Sciences and Computer Engineering, University of Southern Mississippi, Hattiesburg, MS, 39406, United States

## Abstract

Integrative modeling of metagenomic and clinical data can advance the study of host phenotypes, but remains challenged by cross-view heterogeneity, uncertain generalizability, and poor interpretability. We developed SAMECAT (Structure-Aware Metagenomics multi-viEw Contrastive AlignmenT), a structure-aware deep learning framework that integrates species-level shotgun metagenomic profiles with mixed-type clinical covariates through view-specific encoders, clustering-informed contrastive alignment, and adaptive representation fusion. Using two independent Louisiana Osteoporosis Study datasets generated through distinct sequencing and bioinformatics pipelines (development n = 1,990; external evaluation n = 481), we evaluated SAMECAT for bone mineral density prediction at four skeletal sites. SAMECAT consistently outperformed single-view models, naive concatenation, alternative deep learning integration approaches, and established machine learning baselines, with performance gains largely preserved in cross-pipeline external evaluation. To improve biological interpretability, we developed a stability-oriented interpretation workflow that aggregates individually low-magnitude and diffusely distributed feature attributions into structured modules, revealing reproducible site-dependent patterns, coherent functional themes, and representative hub taxa. SAMECAT thus provides a robust and interpretable framework for multi-view metagenomic modeling of microbiome-associated host phenotypes.

## Introduction

The human gut microbiome is increasingly recognized as an important contributor to host physiology and disease [1, 2]. More broadly, the collective metagenome of the human microbiome is often described as a “second genome” because it provides functional capabilities in addition to those encoded by the host genome [3]. Metagenomic data are typically analyzed as relative abundance because raw taxon counts are inherently influenced by sample-specific sequencing depth and are therefore not directly comparable across samples, whereas calibrated absolute load generally requires additional quantitative measurements beyond standard sequencing [4–6]. However, relative abundance data introduce several well-recognized analytical challenges, including compositionality, sparsity, and induced dependence among taxa [5, 7, 8]. These properties complicate microbiome modeling and can violate the assumptions of standard statistical methods, thereby motivating the development of approaches tailored to microbiome data structure [7, 8].

Recent years have seen substantial advances in computational methods for microbiome analysis. For example, MIOSTONE [9] which incorporates taxonomic subordination in its network structure and applies adaptive representation aggregation, has shown overall superior predictive performance compared to numerous tree-aware (with taxonomy or phylogenetic information of microbial taxa leveraged during model construction) and tree-agnostic methods across multiple simulated as well as real microbiome datasets for various disease classification tasks. At the same time, clinical and demographic variables play critical roles in many health outcomes, suggesting that the most informative predictive models may require principled integration of microbiome and non-microbiome information rather than reliance on a single view alone. Accordingly, recent studies have demonstrated that integrating both microbiome profiles and clinical features significantly outperformed the single view models [10, 11].

Despite this progress, most recent efforts to integrate microbiome and clinical information have relied on conventional machine-learning approaches such as random forest and XGBoost [10, 12, 13]. By comparison, relatively few deep-learning studies have explicitly integrated metagenomic relative-abundance profiles with clinical covariates in jointly optimized prediction models [14, 15]. Among the recent peer-reviewed examples, MicroKPNN-MT was designed for host-phenotype prediction using microbiome data together with host metadata [11]. However, its integration strategy is essentially based on direct concatenation, which may be suboptimal for fusing sparse, high-dimensional microbiome profiles with dense, low-dimensional clinical variables. In addition, deep-learning methods for microbiome analysis have largely been developed in classification settings, particularly for disease-state prediction [14, 15]. Yet many host traits of biological and clinical interest are inherently continuous, making regression-based modeling necessary for capturing finer-grained phenotypic variation. Moreover, cross-dataset generalizability remains a critical concern for metagenomic prediction models. Microbiome profiles are sensitive to technical and analytical variation [16], while differences in cohort composition and associated exposures can further shape microbiome profiles and complicate the inference of stable microbiome–host phenotype relationships across datasets [17]. This concern is particularly relevant for flexible machine-learning and deep-learning models, which can capture complex high-dimensional associations but may also learn technical artifacts or unstable feature patterns [18, 19].

Here, we use host bone mineral density (BMD) prediction as a biologically grounded and methodologically stringent testbed. BMD is well suited for this purpose because accumulating evidence supports a gut–bone axis through which the gut microbiome may influence skeletal homeostasis via immune, metabolic, and endocrine pathways [20], while established clinical determinants such as age and sex remain major contributors to BMD variation [21, 22]. We evaluated prediction across four standard skeletal sites—the femoral neck, total hip, total spine, and one-third radius—which represent skeletal regions with differing biomechanical and structural characteristics. These sites also correspond to standard dual-energy X-ray absorptiometry (DXA) regions used in clinical osteoporosis assessment, allowing model performance to be evaluated on outcomes with established diagnostic relevance [23]. Moreover, because our dataset consists predominantly of healthy individuals, BMD variation is subtler than in a case-enriched or clinically extreme cohort, making the prediction task more stringent by requiring recovery of finer-grained inter-individual differences over a narrower dynamic range.

**Figure 1.**
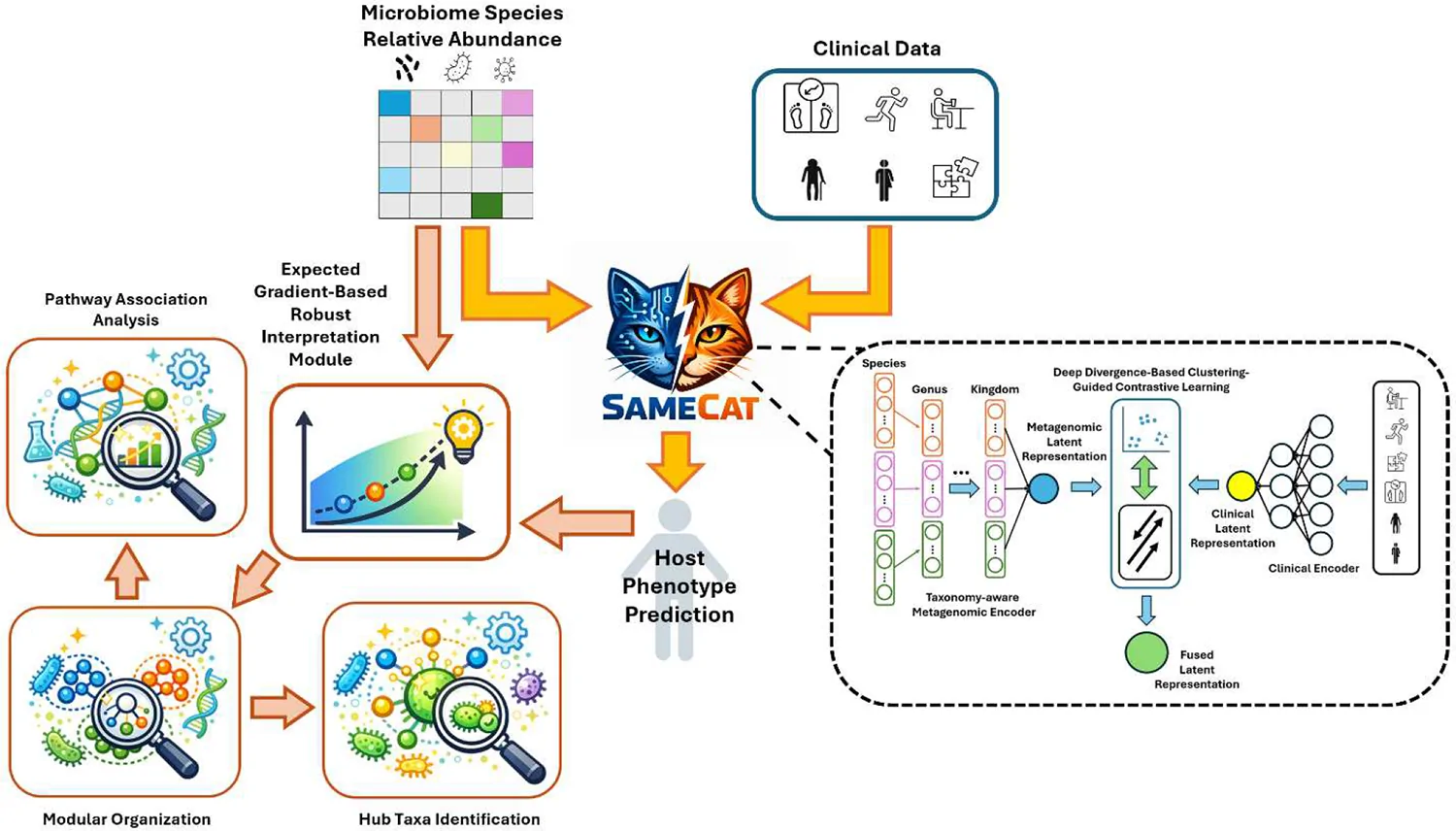
Schematic overview of SAMECAT and the downstream interpretation framework. SAMECAT integrates microbiome species-level relative abundance profiles and clinical variables in a multi-view deep learning framework for host phenotype prediction. Species-level microbiome features are encoded using a taxonomy-aware metagenomic encoder that preserves hierarchical taxonomic structure, whereas clinical covariates are encoded using a Multi-Layer Perceptron (MLP). The resulting modality-specific latent representations are aligned through a clustering–informed contrastive learning strategy and combined into a fused latent representation for downstream prediction. The lower-left section summarizes the post hoc interpretation workflow. Expected Gradients are used to quantify feature-level contributions to model predictions, after which microbial features are organized into attribution-defined modules based on shared predictive contribution patterns. These modules are subsequently used for pathway association analysis and hub taxa identification, enabling biologically structured interpretation of the predictive signals learned by SAMECAT. The SAMECAT icon and the interpretation workflow icons were drafted by ChatGPT version 5.3.

To address these challenges, we developed Structure-Aware Metagenomics multi-viEw Contrastive AlignmenT (SAMECAT) (Fig. 1), a multi-view deep-learning framework that performs structure-aware representation learning for metagenomic relative-abundance profiles and clinical covariates using dedicated view-specific subnetworks, followed by prediction-calibrated fusion through a clustering-informed contrastive learning module. On the intra-cohort held-out testing set, SAMECAT showed superior performance relative to single-view models and other deep-learning integration approaches across skeletal sites and performance metrics. Furthermore, we evaluated SAMECAT against widely used machine-learning baselines, representing the class of methods in which microbiome–clinical integration has been more commonly explored. To directly assess cross-dataset robustness, we adopted a cross-pipeline validation design, in which demographically different cohorts with metagenomic data generated by different companies using substantially different sequencing and bioinformatics workflows were used for training and testing. In this external evaluation, SAMECAT consistently outperformed the machine-learning baselines, demonstrating greater ability to integrate structurally distinct microbiome and clinical features for robust host phenotype prediction.

Beyond prediction, biological interpretation is critical for translating metagenomic modeling results into mechanistic hypotheses and deeper insight into microbiome–host relationships. Previous study has shown that reported microbiome–host phenotype associations can be inconsistent across cohorts, pipelines, and modeling choices [24]. Therefore, predictive accuracy alone does not establish that the learned signals are reproducible or biologically meaningful. Furthermore, although post hoc attribution methods provide feature-level importance estimates [25], our analyses show that microbial attributions can be individually small and diffusely distributed across taxa, limiting direct biological interpretation. A key need is therefore to organize feature-level attributions into reproducible higher-order structures and connect them to functional and taxonomic context. Accordingly, we coupled SAMECAT with an interpretation workflow designed to extract informative patterns from low-magnitude feature attributions. This workflow identifies bootstrap-robust attribution-defined microbial modules, followed by module-wise pathway analysis and hub feature discovery, yielding biologically coherent insights into gut microbiome–bone health associations. Although we applied SAMECAT in the context of BMD, the overall approach is designed for a broader class of microbiome-associated host phenotypes.

Together, our study presents an integrative strategy for multi-view host phenotype prediction and interpretation that achieves accurate and robust performance under stringent prediction settings and across datasets with technical and demographic distribution shifts, while also yielding interpretable results consistent with established biological knowledge, thereby supporting more trustworthy applications of deep learning in microbiome research.

## Results

### SAMECAT Prediction Performance and Comparison with Alternative Approaches

The Louisiana Osteoporosis Study metagenomic data were generated at two independent sequencing facilities: BGI (n = 1,990) and LC (n = 481) (see Methods). Table 1 shows summarized demographic/clinical information for both datasets. Model development, architectural comparisons, and performance benchmarking were conducted using the BGI-derived dataset under repeated internal evaluation (see Methods). The LC-derived dataset, processed using an independent sequencing and bioinformatics pipeline, was reserved exclusively for external evaluation to assess cross-pipeline generalizability. Unless otherwise specified, results reported below refer to internal evaluation on the BGI dataset.

### Performance of SAMECAT Under Different Input Configurations

**Figure 2.**
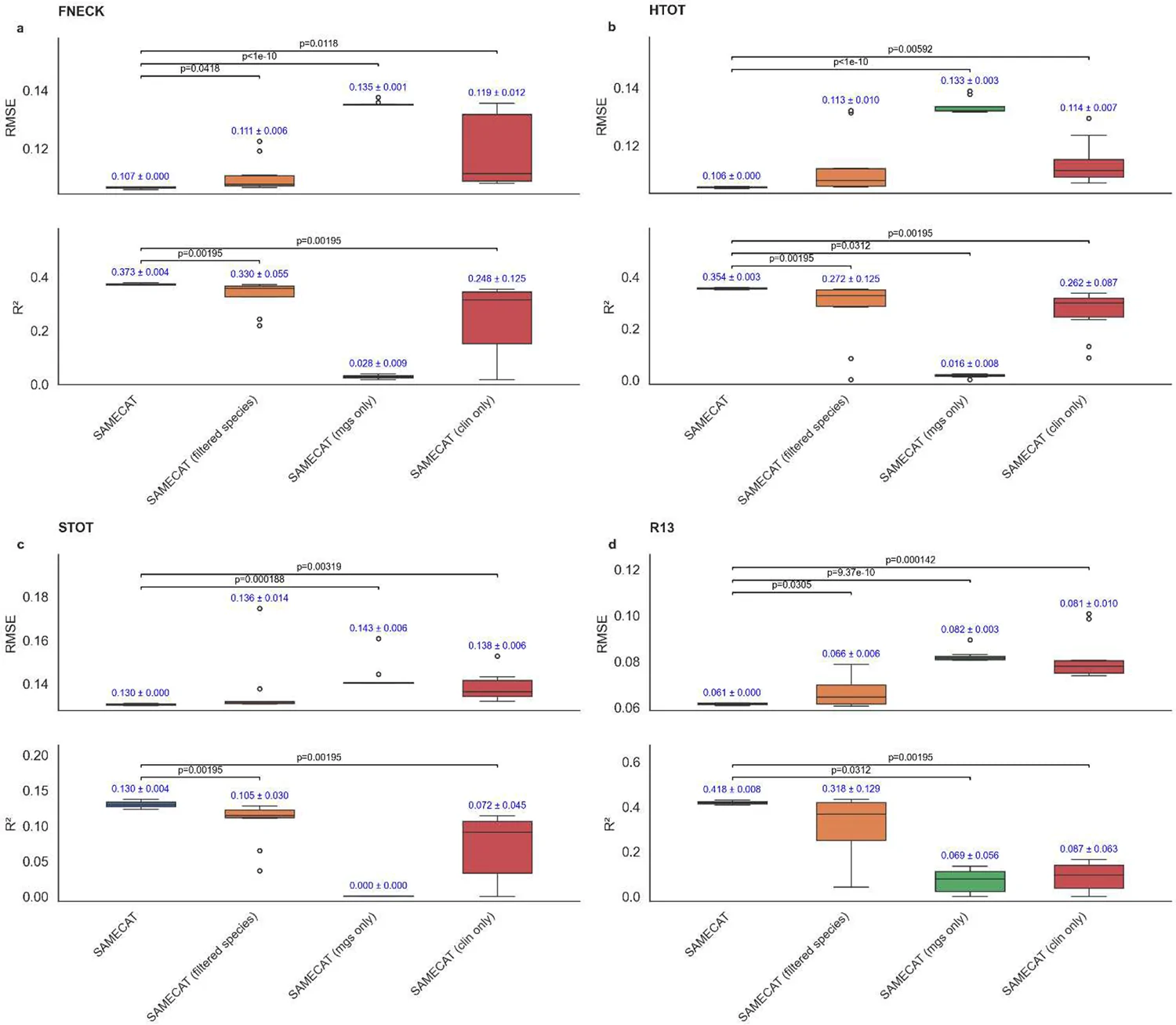
Multi-view integration improves SAMECAT prediction across skeletal sites. Performance of SAMECAT was compared under four input configurations: full multi-view input (clinical variables + microbiome relative abundance [RA]), clinical only, microbiome RA only, and microbiome RA restricted to a prevalence-filtered species set (species prevalence ≥5% in the BGI tuning set). Held-out test performance is shown for **(a)** femoral neck (FNECK), **(b)** total hip (HTOT), **(c)** total spine (STOT), and **(d)** one-third radius (R13) bone mineral density (BMD). evaluated by RMSE (top) and R^2^ (bottom). Boxplots summarize model performance across 10 random training/validation partitions of the BGI tuning set used for hyperparameter tuning. For each partition, the selected optimal hyperparameters were used to retrain the model on the full tuning set and evaluate performance on the held-out BGI testing set. Blue text indicates mean ± SD. P values for significant pairwise comparisons (p<0.05) are shown above the boxplots (paired t-test for RMSE; Wilcoxon signed-rank test for R^2^).

To evaluate the contribution of individual data views and the effectiveness of their integration, we compared the predictive performance of SAMECAT under four input configurations: (i) full multi-view input including clinical variables and microbiome relative abundance (RA), (ii) clinical variables only, (iii) microbiome RA only, and (iv) microbiome RA restricted to a prevalence-filtered species set (prevalence ≥5% in the BGI tuning set, see Methods for detailed information on data partition and model training/evaluation designs) [27]. Model performance was assessed on held-out BGI testing data using RMSE (Root Mean Squared Error) and correlation-based $R^{2}$, defined as the squared Pearson correlation between predicted and observed BMD, across femoral neck (FNECK), total hip (HTOT), total spine (STOT), and one-third radius (R13) (Fig. 2a–d).

Across all skeletal sites, SAMECAT with full multi-view input consistently achieved the best predictive performance, yielding the lowest RMSE and highest $R^{2}$ relative to all other configurations. On the other hand, models trained using either clinical variables only or microbiome RA only showed substantially degraded performance, with microbiome-only models exhibiting near-zero $R^{2}$ in several sites.

Importantly, the single-view SAMECAT variants correspond to established neural network architectures specialized for individual data structure. The microbiome RA–only SAMECAT is functionally equivalent to a taxonomy-aware MIOSTONE network [9] optimized for sparse and high-dimensional microbial abundance profiles, whereas the clinical-only SAMECAT reduces to a standard multilayer perceptron (MLP) operating on dense, low-dimensional clinical covariates. While each architecture captures view-specific signals, their isolated application results in limited predictive power.

Restricting microbiome input to a prevalence-filtered species set led to a clear reduction in predictive performance relative to the full multi-view model. Nevertheless, multi-view SAMECAT trained on the prevalence-filtered microbiome input consistently outperformed the microbiome-only model and achieved nominally better performance than the clinical-only model across skeletal sites (see Supplementary Fig. S1), indicating that even a partially degraded microbiome representation contributes non-redundant predictive information when integrated with clinical features. Together, these findings suggest that SAMECAT effectively captures joint predictive signals from sparse microbiome and dense clinical data, while also benefiting from the retention of low-prevalence taxa.

### Comparison with Alternative Deep Learning Integration Strategies

**Figure 3.**
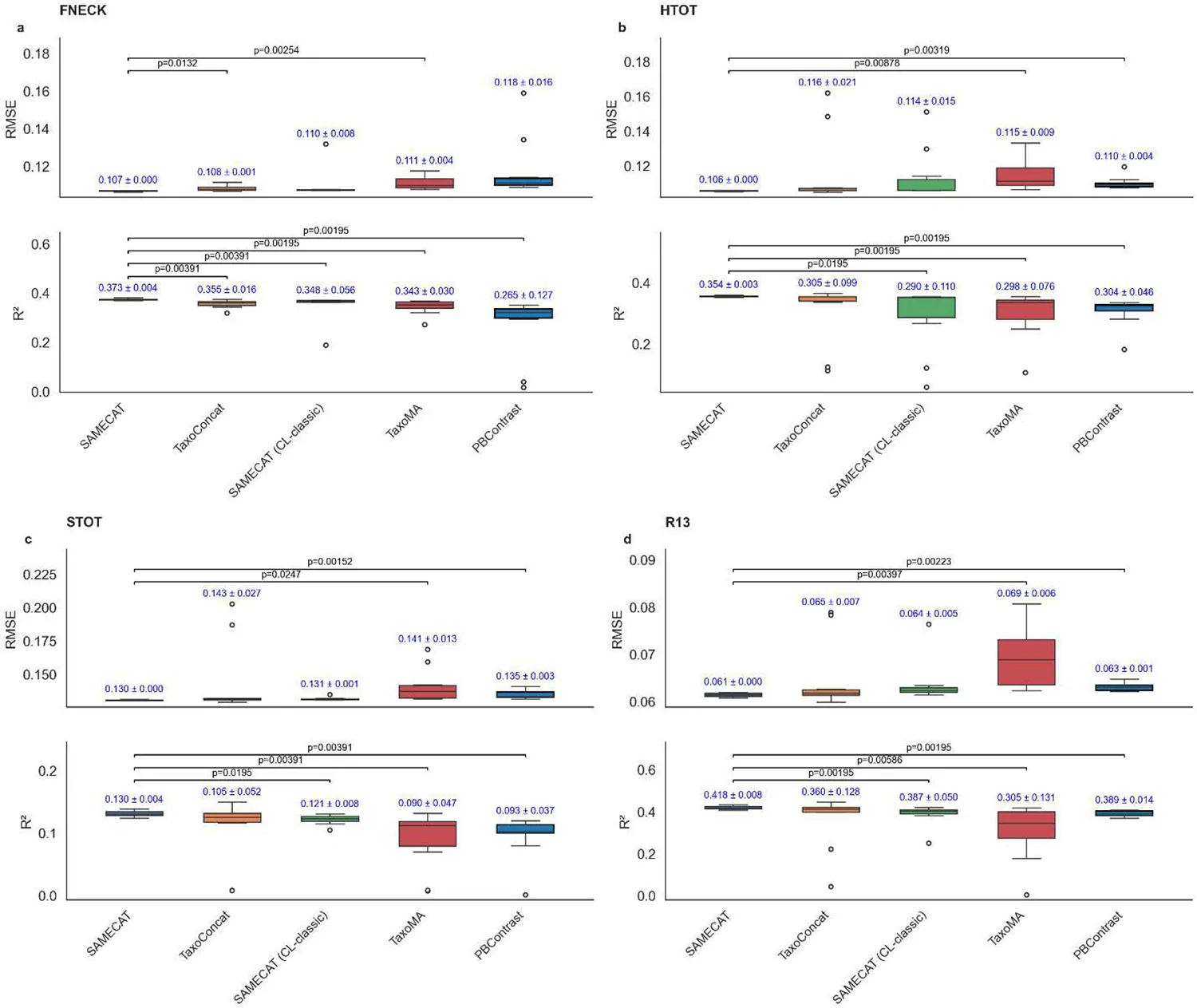
Comparison of SAMECAT with alternative deep-learning integration strategies. SAMECAT was compared with several alternative deep-learning and representation-learning approaches for integrating clinical and microbiome data, including direct concatenation of modality-specific latent representations (TaxoConcat), a SAMECAT variant using classic contrastive learning, a manifold-alignment–based integration strategy (TaxoMA), and a principal balance–based approach (PBContrast). Predictive performance on held-out test data is shown for **(a)** femoral neck (FNECK), **(b)** total hip (HTOT), **(c)** total spine (STOT), and **(d)** one-third radius (R13) BMD, evaluated using RMSE (top) and R^2^ (bottom). Boxplots summarize model performance across 10 random training/validation partitions of the BGI tuning set used for hyperparameter tuning. For each partition, the selected optimal hyperparameters were used to retrain the model on the full tuning set and evaluate performance on the held-out BGI testing set. Blue text indicates mean ± SD for each method. P values for significant pairwise comparisons (p < 0.05) are shown above the boxplots; paired t-tests were used for RMSE and Wilcoxon signed-rank tests were used for R^2^.

To determine whether SAMECAT’s performance gains are attributable to its integration strategy rather than to multi-view input alone, we compared SAMECAT against several alternative deep learning and representation-learning approaches for integrating clinical and microbiome data (Fig. 3a–d). These alternatives include: (i) direct concatenation of latent representations learned by view-specific subnetworks; (ii) SAMECAT with classic contrastive learning applied to latent representations without negative sampling [28]; (iii) a multi-view integration strategy based on manifold alignment (we denote it as TaxoMA) [29–31], in which subject-level correspondence between views was first learned and subsequently aggregated in latent space; and (iv) a principal balance–based approach (we named it PBContrast) [32, 33], in which metagenomic relative abundance data were transformed into isometric log-ratio principal balances prior to model fitting. For PBContrast, balances were fed into an MLP directly without taxonomy-aware encoding, and the same multi-view integration strategy as SAMECAT was applied during training (for more details on TaxoMA and PBContrast, see Supplementary Methods).

Across all skeletal sites, SAMECAT consistently achieved the lowest RMSE and highest R^2^ on the held-out testing set. Direct concatenation of latent representations resulted in inferior performance, indicating that naive fusion of modality-specific embeddings is insufficient to capture the cross-view relationship structure that is essential for the prediction tasks. The SAMECAT variant incorporating a conventional contrastive learning objective (without negative sampling) also underperformed the original model. In our implementation, negative pairs were drawn only from subjects assigned to different clusters according to the deep divergence-based clustering (DDC) [34] (see Methods). By restricting repulsion to cross-cluster pairs, the contrastive objective preserves within-cluster cohesion while promoting cross-view consistency [35], which potentially led to the observed performance advantage.

The manifold-alignment–based integration strategy also underperformed SAMECAT across skeletal sites. Although this approach explicitly aligns subject-level geometric structure between views (see Supplementary Methods), its multi-view fusion is learned solely from input-space correspondence. In contrast, SAMECAT performs representation integration under supervision from the downstream prediction task. The consistent advantage of SAMECAT therefore highlights the importance of prediction-calibrated multi-view integration, rather than relying on unsupervised geometric alignment alone.

For the principal balance–based approach (PBContrast), when PBContrast was constructed using a prevalence-filtered species set and compared against SAMECAT trained on the full species set, SAMECAT demonstrated significantly superior predictive performance across skeletal sites. This again implies predictive signal lost under reduced feature availability and that balance-based compositional transformation was unable to compensate for this loss.

To isolate the effect of representation learning method from feature availability, we additionally compared SAMECAT and PBContrast under a fully controlled setting in which both models were trained using the same prevalence-filtered species set (Supplementary Fig. S2). Under this condition, no statistically significant differences in predictive performance were observed across skeletal sites. Interestingly, PBCantrast also gave nominally better performance than clinical-only SAMECAT, indicating that the integration strategy of SAMECAT remained effective under restricted feature availability across different microbial relative-abundance modeling approaches. Importantly, despite the similarity in prediction performance, key distinctions remain at the representation and interpretability levels between SAMECAT and PBContrast. SAMECAT enables direct feature-wise attribution aligned with microbial taxonomy and preserves hierarchical biological structure within the learned representation. In contrast, PBContrast produces balance-level attributions defined over log-contrast coordinates; while these can be algebraically decomposed to recover species contributions, the representation does not explicitly encode taxonomic hierarchy or biological structure. Furthermore, SAMECAT operates as an end-to-end learning framework applied directly to species-level relative abundance data, whereas PBContrast requires explicit construction of a balance basis prior to model training. This additional preprocessing step introduces methodological complexity and may reduce portability across datasets with differing species compositions.

Collectively, these findings demonstrate that the cluster-informed and prediction-aligned integration of SAMECAT contributed to effective fusion of distinct structural characteristics carried by clinical and microbiome data, yielding consistent improvements in BMD prediction across different skeletal sites compared to alternative integration strategies.

### Comparison with Conventional Machine Learning Approaches

**Figure 4.**
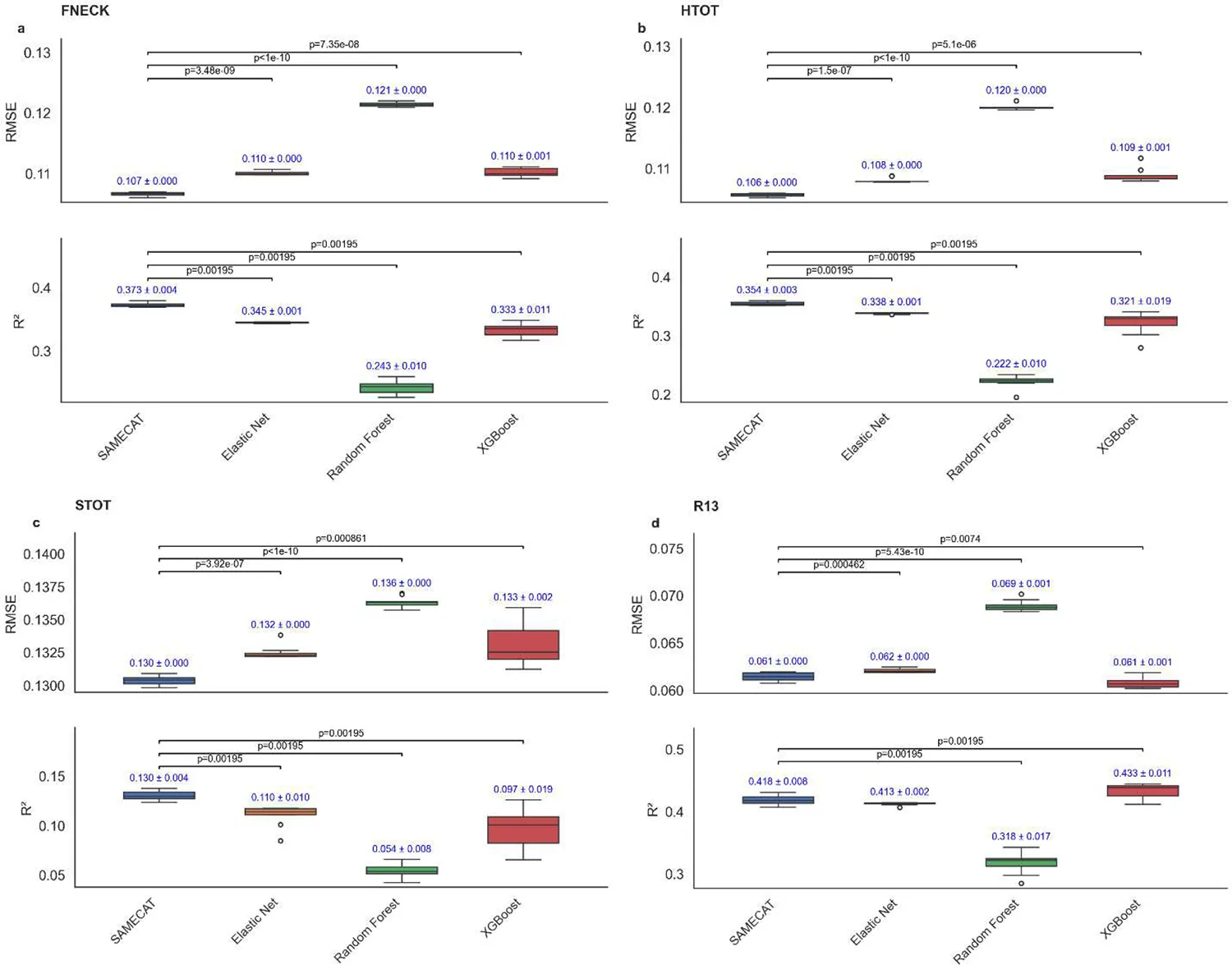
Comparison of SAMECAT with established machine-learning approaches across skeletal sites. SAMECAT was compared with established machine-learning methods, including Elastic Net, Random Forest, and XGBoost, for prediction of **(a)** femoral neck (FNECK), **(b)** total hip (HTOT), **(c)** total spine (STOT), and **(d)** one-third radius (R13) BMD, evaluated using RMSE (top) and R^2^ (bottom). Boxplots summarize model performance across 10 random training/validation partitions of the BGI tuning set used for hyperparameter tuning. For each partition, the selected optimal hyperparameters were used to retrain the model on the full tuning set and evaluate performance on the held-out BGI testing set. Blue text indicates mean ± SD for each method. P values for significant pairwise comparisons (p < 0.05) are shown above the boxplots; paired t-tests were used for RMSE and Wilcoxon signed-rank tests were used for R^2^.

To place the performance of SAMECAT in broader context, we compared it against established machine learning approaches, including elastic net, random forest, and XGBoost (Fig. 4a–d). Tree-based ensemble methods have been shown to frequently match or outperform deep neural networks on tabular datasets, particularly in moderate-sample settings [36, 37], rendering them stringent comparators. Elastic net was additionally included as a regularized linear baseline that induces sparsity through variable selection, stabilizes coefficient estimation under multicollinearity, and promotes a grouping effect among correlated predictors [38]. These properties are particularly relevant in high-dimensional microbiome studies, where compositional constraints induce strong dependency structures and correlations among relative abundance features [5, 39].

Across FNECK, HTOT, and STOT, SAMECAT significantly outperformed elastic net, random forest, and XGBoost in pairwise comparisons for both performance metrics. The consistency of these findings across different weight-bearing skeletal sites indicates that conventional machine learning models, whether linear regularized or tree-based ensemble methods, are limited in capturing the joint predictive signals underlying microbiome composition and clinical covariates.

For R13, XGBoost achieved significantly lower RMSE and higher R^2^ than SAMECAT. Elastic net showed comparable R^2^ but significantly higher RMSE, whereas random forest yielded the weakest results. Although gradient boosting achieved the strongest performance at the largely non–weight-bearing R13 site, the observed differences relative to SAMECAT were modest in scale.

To clarify whether the observed performance gains were attributable to deep model capacity rather than structured multi-view integration, we evaluated machine learning approaches under a clinical-only input setting (Supplementary Fig. S3). Clinical covariates constitute dense, low-dimensional tabular data. Evaluating model performance in this single-view setting therefore provides a controlled context in which conventional machine learning methods serve as strong baselines relative to generic neural networks. Indeed, conventional machine learning models outperformed the SAMECAT clinical-only variant (which reduces to an MLP). This finding demonstrates that the dataset itself does not intrinsically favor deep learning architectures. Moreover, when machine learning models were provided with both clinical variables and species-level microbial relative abundance as input, their performance frequently deteriorated relative to their clinical-only counterparts. This pattern suggests overfitting and ineffective utilization of high-dimensional microbiome features.

Finally, we compared the full SAMECAT model (clinical + microbiome inputs) against machine learning models trained using clinical covariates alone (Supplementary Fig. S4). Although tree-based and regularized linear methods showed strong performance when restricted to dense clinical features, the full SAMECAT model exceeded their predictive performance at femoral neck, total hip, and total spine, yielding consistent gains beyond those achievable with clinical covariates alone. Together, these findings demonstrate that SAMECAT’s advantage arises from effective cross-view integration rather than architectural complexity, enabling it to leverage complementary microbiome–clinical information in a manner that the machine learning alternatives fail to capture.

### External Evaluation Across Independent Sequencing and Bioinformatics Pipelines

We next evaluated whether the predictive advantage of SAMECAT generalizes across independently generated metagenomic datasets. Using the LC-derived cohort as an external test set, models were trained exclusively on the BGI tuning data with hyperparameters fixed from internal evaluation and applied directly to LC samples without retraining (see Methods). The LC and BGI datasets differ in sequencing process and taxonomic profiling workflows (see Methods), factors known to introduce systematic biases in metagenomic abundance estimation [16]. Species-level taxonomic profile comparisons are summarized in Supplementary Table S1. This design therefore constitutes a stringent cross-pipeline generalization test.

For external evaluation, we focused comparisons on the established machine learning baselines (i.e., elastic net, random forest, and XGBoost). These baselines represent linear and nonlinear machine learning paradigms that operate directly in the original feature space without explicit structural encoding or learned representation alignment.

**Figure 5.**
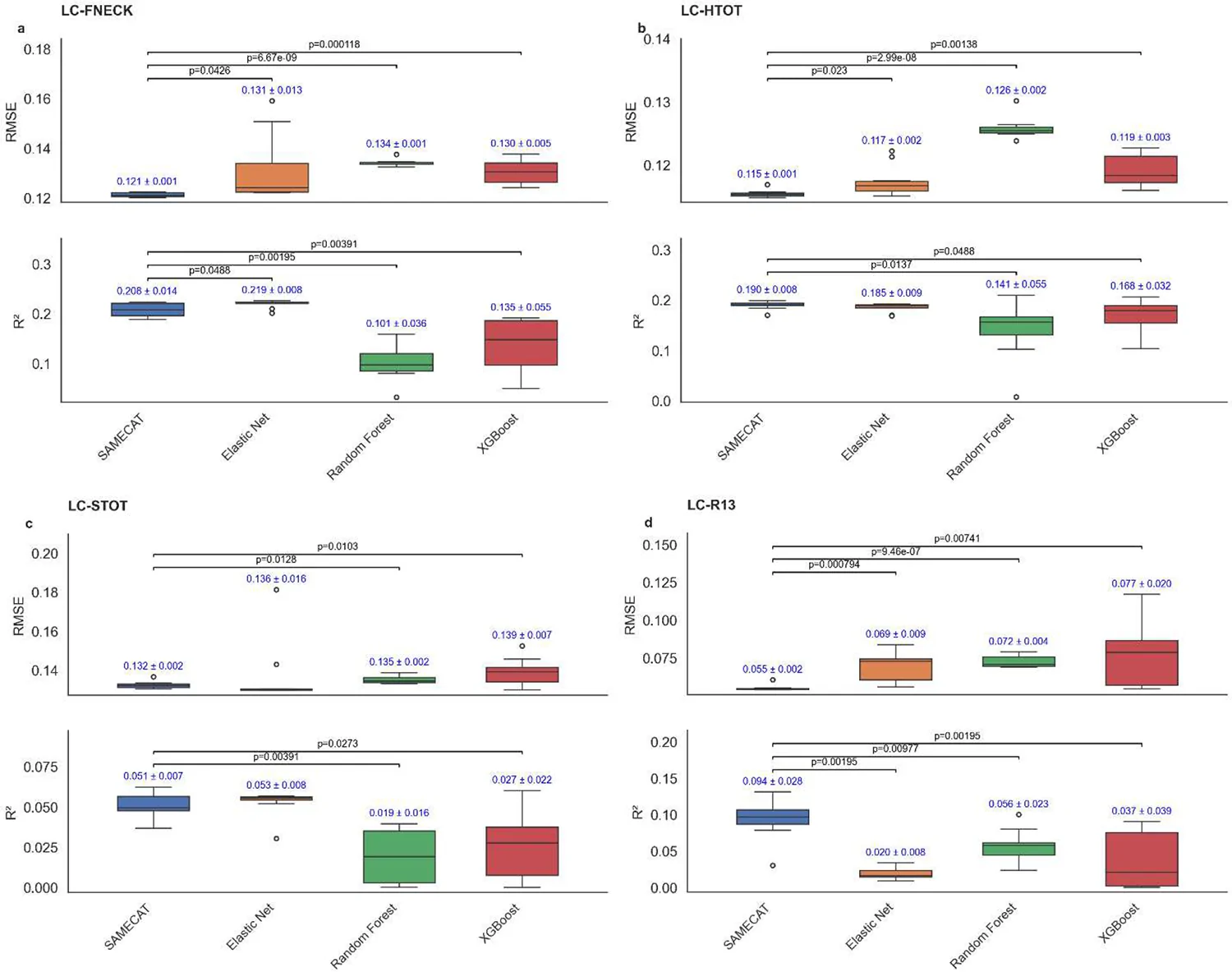
Cross-pipeline external evaluation of SAMECAT and comparator models. External evaluation was performed by applying models trained on the BGI-derived tuning data directly, without retraining, to the independent LC-derived data. Predictive performance is shown for **(a)** femoral neck (FNECK), **(b)** total hip (HTOT), **(c)** total spine (STOT), and **(d)** one-third radius (R13) BMD, evaluated using RMSE (top) and R^2^ (bottom). Methods compared include SAMECAT, Elastic Net, Random Forest, and XGBoost. Boxplots summarize the 10 external-testing performance values obtained on the LC data from the 10 models trained on the BGI tuning set using the best hyperparameter configuration selected from the corresponding training/validation data partition. Blue text indicates mean ± SD for each method. P values for significant pairwise comparisons (p<0.05) are shown above the boxplots; paired t-tests were used for RMSE and Wilcoxon signed-rank tests were used for R^2^.

In Fig. 5, across skeletal sites, SAMECAT remained highly competitive and exhibited the most consistent overall robustness under cross-pipeline perturbation. For total hip and one-third radius, SAMECAT achieved both the lowest mean RMSE and the highest mean R^2^ among all methods, indicating superior cross-pipeline generalization at these sites. For femoral neck, SAMECAT yielded the lowest mean RMSE, reflecting the best absolute predictive accuracy, whereas Elastic Net showed a modest advantage in mean R^2^. A similar pattern was observed for total spine, where SAMECAT again achieved the lowest mean RMSE, while Elastic Net showed a slightly higher mean R^2^. In contrast, random forest and XGBoost generally exhibited weaker and less stable performance across skeletal sites.

In addition to technical differences, the LC and BGI cohorts differed in demographic composition, including sex distribution and age structure (Table 1). Notably, the LC dataset consisted exclusively of male participants, whereas the BGI dataset included both sexes. Because age and sex are well-established determinants of BMD variation [21, 22], these differences introduce additional distributional shifts beyond metagenomic pipeline heterogeneity. All models incorporated identical clinical covariates during training and evaluation. The persistence of SAMECAT’s performance advantage under these compound shifts indicates that its structure-aware and prediction-calibrated multi-view representations capture stable predictor-outcome relationships rather than dataset-specific artifacts. Importantly, no domain adaptation or retraining was performed on the LC dataset, indicating that the observed generalization reflects intrinsic robustness of the learned representation.

Overall, these findings demonstrate that SAMECAT’s advantage extends beyond internal benchmarking and persists under stringent cross-pipeline validation. Next, we interrogated the feature-level attribution structure underlying SAMECAT’s predictions. We assessed whether the learned attribution patterns exhibit structured and reproducible organization aligned with prior biological findings, thereby linking predictive generalizability with interpretive fidelity.

### Model Interpretation and Attribution Analysis

As an initial step in interpreting the learned model, we examined the relative contribution of each view through the fusion weights (normalized via SoftMax function, ranging from 0 to 1) of the multi-view integration module (see Supplementary Fig. S5). Across tuning-selected hyperparameter settings, the latent representation derived from metagenomic relative abundance frequently received fusion weights at or above 0.5, indicating that the microbiome latent representation contributed at least comparably to the clinical representation in generating BMD predictions for SAMECAT.

Wilcoxon signed-rank tests comparing microbiome-derived fusion weights against the null value of 0.5 demonstrated statistically significant predominance of the microbiome representation for total hip (p = 0.037) and total spine (p = 0.019). For femoral neck and one-third radius, microbiome fusion weights exceeded 0.5 in 7/10 and 6/10 hyperparameter settings, respectively, although these trends did not reach statistical significance. Notably, no skeletal site exhibited consistent dominance of the clinical representation across different hyperparameter configurations of SAMECAT.

These findings indicate that the learned predictive function materially incorporates microbial relative abundance signals rather than relying primarily on clinical covariates. Given this substantial contribution of the microbiome-derived representation, we next quantified feature-level attributions using Expected Gradients (EG) [25], a gradient-based attribution method designed to provide stable and reference-aware estimates of input importance (see Methods).

We then examined whether the resulting attribution patterns reveal organized and biologically interpretable structure across skeletal sites.

### Attribution-Driven Module Discovery via Spectral Clustering

While EG quantifies feature-level contributions to BMD prediction, individual attributions can be small in magnitude (see Supplementary Fig. S6). Moreover, these attributions alone do not reveal whether predictive signals are organized into higher-order, structured patterns. Therefore, we next applied attribution-driven spectral clustering to the EG matrix for each skeletal site to determine whether SAMECAT’s learned attribution landscape exhibits modular organization. Species assigned to the same module on the basis of similarity in their EG profiles therefore exhibit concordant attribution patterns, suggesting shared relationships with BMD and potentially reflecting coordinated functional influences on skeletal biology.

Briefly, subject-level EG scores were first scale-normalized using a signed-log transformation (see Methods) to reduce the influence of extreme values while preserving directional information. Feature–feature similarity was then computed using cosine similarity across subjects, yielding an affinity graph that captures similarity in predictive contribution profiles rather than taxonomic proximity. Spectral clustering was performed on the normalized graph Laplacian to identify modules of microbial features exhibiting coherent attribution patterns. Because spectral clustering outcomes depend on hyperparameter choices, we implemented a bootstrap-based stability assessment and composite stability criterion to select robust configurations (see Methods). This procedure ensured that identified modules were reproducible under subject resampling and not artifacts of specific parameter settings.

**Figure 6.**
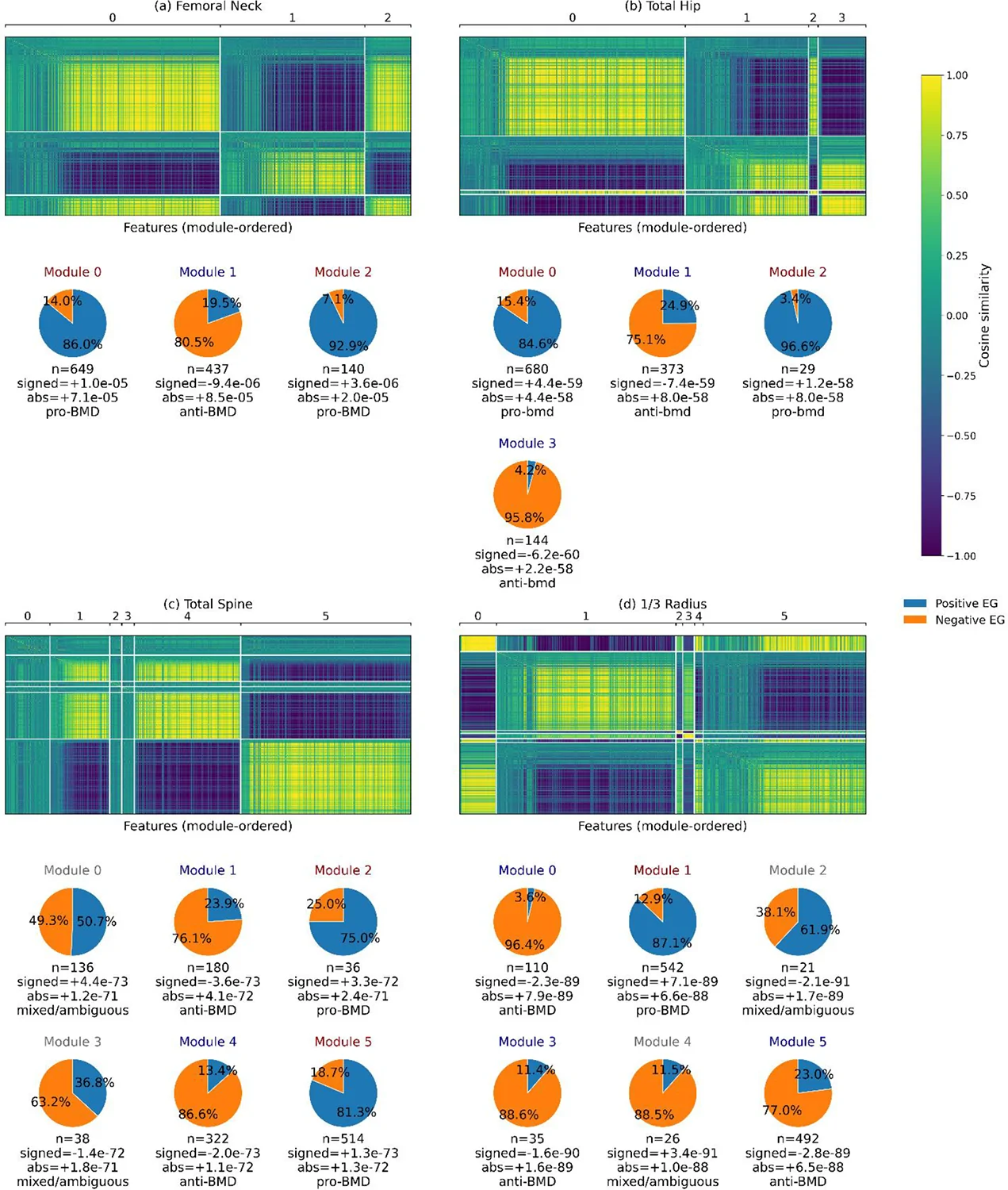
Attribution-driven microbial modules identified from feature-level EG similarity. Heatmaps show feature–feature cosine similarity matrices computed from subject-level Expected Gradient (EG) attribution profiles for models predicting BMD at the **(a)** femoral neck, **(b)** total hip, **(c)** total spine, and **(d)** one-third radius. EG values were first transformed using a scale-normalized signed-log function (see Methods), and spectral clustering was applied to identify groups of microbial features with coherent attribution patterns. Features are ordered by module assignment, and block-diagonal structure indicates within-module attribution coherence. Pie charts summarize the directional composition of each module, showing the proportions of features with positive EG (blue) and negative EG (orange). For each module, n denotes the number of features, and the reported signed and absolute values summarize the mean signed EG and mean absolute EG, respectively. Modules were classified as pro-BMD, anti-BMD, or mixed/ambiguous according to their dominant attribution direction (i.e., whether at least 75% of species in the module share the same attribution sign as the module mean attribution).

In Fig. 6, across all four skeletal sites, clear block-diagonal structures were observed in the feature–feature cosine similarity matrices, indicating strong within-module attribution coherence. However, the number of stable modules differed by site: three modules for femoral neck, four for total hip, and six modules each for total spine and one-third radius. This variation indicates that the structure of microbiome-derived predictive signals may differ between skeletal sites.

Importantly, directional coherence within modules was also evident. For femoral neck and total hip, all identified modules exhibited clear pro- or anti-BMD attribution patterns, with at least 75% of species in each module sharing the same attribution sign as the module mean. For total spine and one-third radius, four of six modules per site demonstrated similarly consistent directional structure, whereas the remaining modules showed mixed or attenuated patterns. These results indicate that SAMECAT’s diffusely distributed predictive signals are successfully organized into reproducible microbial groups with coordinated positive or negative contributions to BMD prediction.

### Cross-Site Structure of Module–Pathway Associations

To translate attribution-driven microbial modules into interpretable biological functions, we performed module-wise pathway association analysis using stratified pathway abundance and pathway coverage profiles reconstructed from shotgun metagenomic data via HUMAnN2 [40]. For each site-specific module, pathway abundance was aggregated across microbial features assigned to the module, and Spearman correlations (ρ) were computed between module-level Expected Gradient (EG) signals and pathway abundance across subjects. Bootstrap resampling was used to assess stability of each module–pathway association, and pathway coverage was incorporated to prioritize structurally well-represented pathways, as detailed in the Pathway Relevance Score (PaRS) framework in Methods. In Supplementary Fig S7, we illustrated the top-ranked (top 10 in each module of each site) module–pathway pairs using effect–stability plots. Specifically, we plotted their absolute correlation magnitude (|Spearman ρ|) against bootstrap stability (see Methods), with point size proportional to mean pathway coverage. The effect–stability plots showed that the top-ranked pathway-module pairs predominantly occupy the high-confidence region, characterized by large |ρ| and high stability, indicating that the strongest functional signals were reproducible across resamples and therefore suitable for downstream biological interpretation.

**Figure 7.**
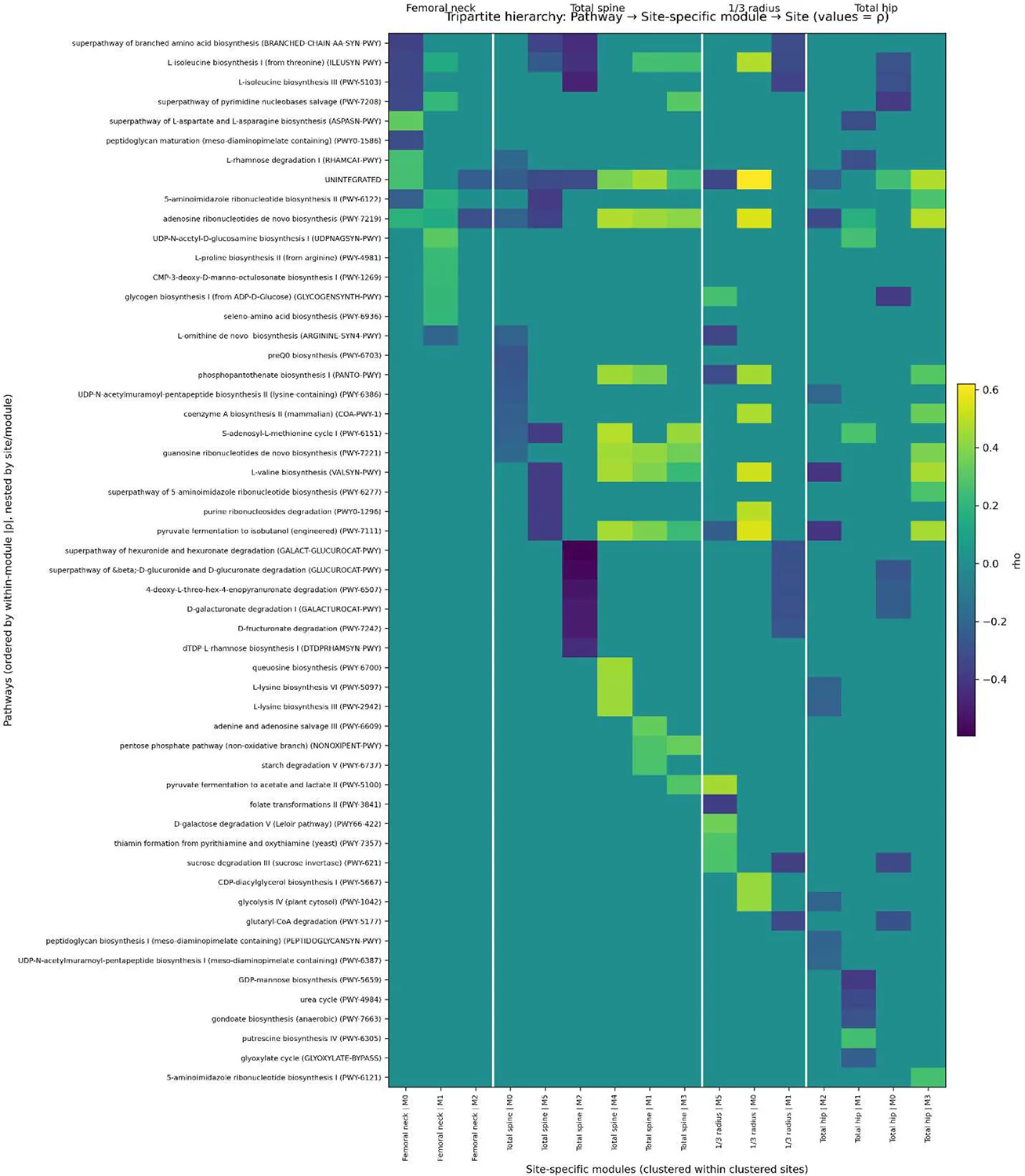
Module-level pathway associations across skeletal sites (top 10 in each module of each site). Tripartite heatmap summarizing associations between microbial pathways, attribution-defined modules and skeletal sites in SAMECAT. For each site-specific module, pathway abundance was aggregated across assigned microbial features from stratified HUMAnN2 profiles, and Spearman correlations (ρ) were computed between module-level Expected Gradient (EG) signals and pathway abundance across subjects. Pathways were prioritized using the Pathway Relevance Score framework (see Methods), which incorporates effect size, pathway coverage and bootstrap stability. Columns represent site-specific modules grouped by skeletal site; module numbers are site-specific and do not indicate cross-site correspondence. Skeletal sites and modules within each site were ordered by hierarchical clustering of pathway association profiles. Pathways in the rows were ranked by decreasing absolute Spearman correlation (|ρ|) and appended sequentially across clustered modules, with duplicate pathways removed after first occurrence to preserve global uniqueness. Each cell shows the signed pathway–module Spearman correlation.

We summarized top-ranked module–pathway relationships using a tripartite hierarchy (Pathway → site-specific module → skeletal site; Fig. 7). Columns correspond to site-specific modules; module numbering reflects discovery order within each site and does not imply cross-site correspondence. Skeletal sites were ordered by hierarchical clustering of site-level pathway association profiles using Pearson correlation distance (1−r) with average linkage. Site-level pathway association values were defined by aggregating module-level pathway correlations within each site, selecting for each pathway the module exhibiting the largest absolute association magnitude (|Spearman ρ|) and retaining its signed value, such that sites with similar functional association patterns are displayed adjacently. Within each skeletal site, modules were likewise ordered by hierarchical clustering of their pathway association vectors using Pearson correlation distance (1−r) and average linkage, so that modules with similar functional signatures appear adjacently rather than in numeric order.

Pathway rows were constructed in a nested manner by iterating over clustered sites and clustered modules within each site; for each module, pathways were ranked by decreasing absolute Spearman correlation (|ρ|) and appended sequentially, with duplicate pathways omitted after their first occurrence to enforce global uniqueness. Each heatmap cell encodes the signed Spearman correlation (ρ) between module-level EG signal and pathway abundance, enabling simultaneous inspection of site specificity, module specificity, and directional concordance of functional signals.

Across skeletal sites, the tripartite summary plot revealed heterogeneous but structured patterns of pathway–module associations. Femoral neck BMD showed the sparsest association pattern, with relatively few pathways demonstrating strong correlations with module-level attribution signals. In contrast, total spine displayed denser association patterns, with a larger number of pathway–module pairs and individual pathways more frequently correlating with multiple attribution modules. One-third radius and total hip exhibited intermediate patterns.

Anatomically, skeletal sites differ in both bone compartment composition and mechanical loading environment [41–48]. The lumbar spine is enriched in trabecular bone, which exhibits higher metabolic activity and higher turnover rate compared with cortical bone [41]. Therefore, trabecular-rich skeletal regions may be more sensitive to systemic metabolic signaling processes that can be influenced by the gut microbiome [20]. This anatomical characteristic may contribute to the relatively dense pathway–module association patterns observed for spine BMD. Furthermore, because total spine BMD reflects an aggregate measurement across several lumbar vertebrae, biomechanical influences acting at individual vertebrae may be partially averaged across the region, potentially reducing the impact of localized biomechanical “noise” on the detection of systemic physiological influences.

The femoral neck contains a substantial cortical proportion and functions as a major load-transmitting structure of the proximal femur during weight-bearing activities [43, 44, 46–48]. Because femoral neck BMD reflects bone mineral density within a relatively localized anatomical region that experiences strong biomechanical loading, local mechanical determinants may contribute substantially to variation in BMD at this site. In addition, the higher cortical bone proportion of the femoral neck is generally associated with lower bone turnover and metabolic activity compared with trabecular-rich skeletal regions [41]. Together, these factors may potentially attenuate detectable pathway–module correlations associated with microbial functional signals.

Total hip BMD integrates signals from multiple anatomical subregions—including the femoral neck, trochanteric, and intertrochanteric regions—resulting in a composite cortical–trabecular phenotype [46]. Moreover, like the total spine, the aggregate nature of the total hip measurement potentially averages localized biomechanical influences and facilitates the detection of systemic physiological signals, as reflected by the broader pathway–module associations observed.

The one-third radius represents a predominantly cortical and largely non–weight-bearing skeletal site [41, 49]. As shown in Fig. 7, while only a subset of attribution modules showed strong pathway-level correspondence at this site, those informative modules exhibited dense pathway–module associations. Although cortical bone typically exhibits lower metabolic activity than trabecular bone, these association patterns may reflect the integration of systemic metabolic influences over more extended timescales. Furthermore, the absence of substantial weight-bearing mechanical loading likely reduces biomechanics-related variation in BMD, potentially allowing microbiome-linked systemic signals to be captured more clearly within functionally coherent modules. The remaining modules may reflect predictive microbial structure that is mediated through host processes not directly resolved by metagenomic pathway profiles.

### Biological Themes Emerging from Attribution-Linked Pathways

Inspection of the pathway categories associated with attribution modules reveals several recurring functional themes across skeletal sites. These include pathways involved in amino acid biosynthesis (e.g., *L-valine biosynthesis* [VALSYN-PWY], *L-isoleucine biosynthesis* [ILEUSYN-PWY], *L-proline biosynthesis II* [PWY-4981]), nucleotide metabolism (e.g., *adenosine ribonucleotides de novo biosynthesis* [PWY-7219], *guanosine ribonucleotides de novo biosynthesis* [PWY-7221]), central carbon metabolism (e.g., *glycolysis IV* [PWY-1042], *pentose phosphate pathway* (*non-oxidative branch*) [NONOXIPENT-PWY]), and cofactor and one-carbon metabolic processes (e.g., *folate transformations II* [PWY-3841], *S-adenosyl-L-methionine cycle* [PWY-6151]).

Microbiota can reshape host amino-acid availability by efficiently metabolizing intestinal amino acids, creating a mechanistic link between microbial amino-acid metabolic capacity and host nutrient pools [50]. In humans, circulating amino-acid profiles—including branched-chain amino acids (e.g., valine, isoleucine)—have been associated with longitudinal BMD decline and future fracture risk, supporting the relevance of systemic amino-acid availability to bone outcomes [51]. At the cellular level, amino-acid supply is a key constraint on osteoblast differentiation and matrix production: osteoblast differentiation increases demand for proline, and genetic ablation of the proline transporter SLC38A2 in osteoblasts limits osteoblast differentiation and bone formation [52].

Gut microbial purine metabolism may influence bone outcomes indirectly by modulating systemic purine metabolite homeostasis (including serum uric acid and related purine metabolites) and by providing luminal ATP cues that promote Th17 differentiation, thereby engaging osteoclastogenic inflammatory programs and inflammatory bone resorption [53–57].

The human gut microbiota encodes broad-spectrum B-vitamin biosynthesis potential (including folates), and commensals have been discussed as vitamin suppliers to the host; folate is central to one-carbon transfer and is linked to S-adenosylmethionine (SAM) methylation potential and nucleotide biosynthesis [58, 59]. Disruption of one-carbon metabolism—often indexed by hyperhomocysteinemia and B-vitamin/folate status—has been linked to altered bone formation/remodeling and fracture risk in experimental and population studies, and SAM-dependent methylation has been shown to regulate osteoblast differentiation through Runx2-controlled gene expression programs [60, 61].

Finally, central carbon metabolism and fermentation pathways can influence host bone through microbial metabolite production, particularly short-chain fatty acids (SCFAs) generated by fermentation of dietary fiber through glycolysis and the pentose-phosphate pathway [62]. In mice, SCFAs have been shown to increase bone mass and protect against pathological bone loss, with mechanistic links to osteoclast differentiation and immune-metabolic reprogramming [63].

### Hub Microbial Feature Summary Across Skeletal Sites

**Figure 8.**
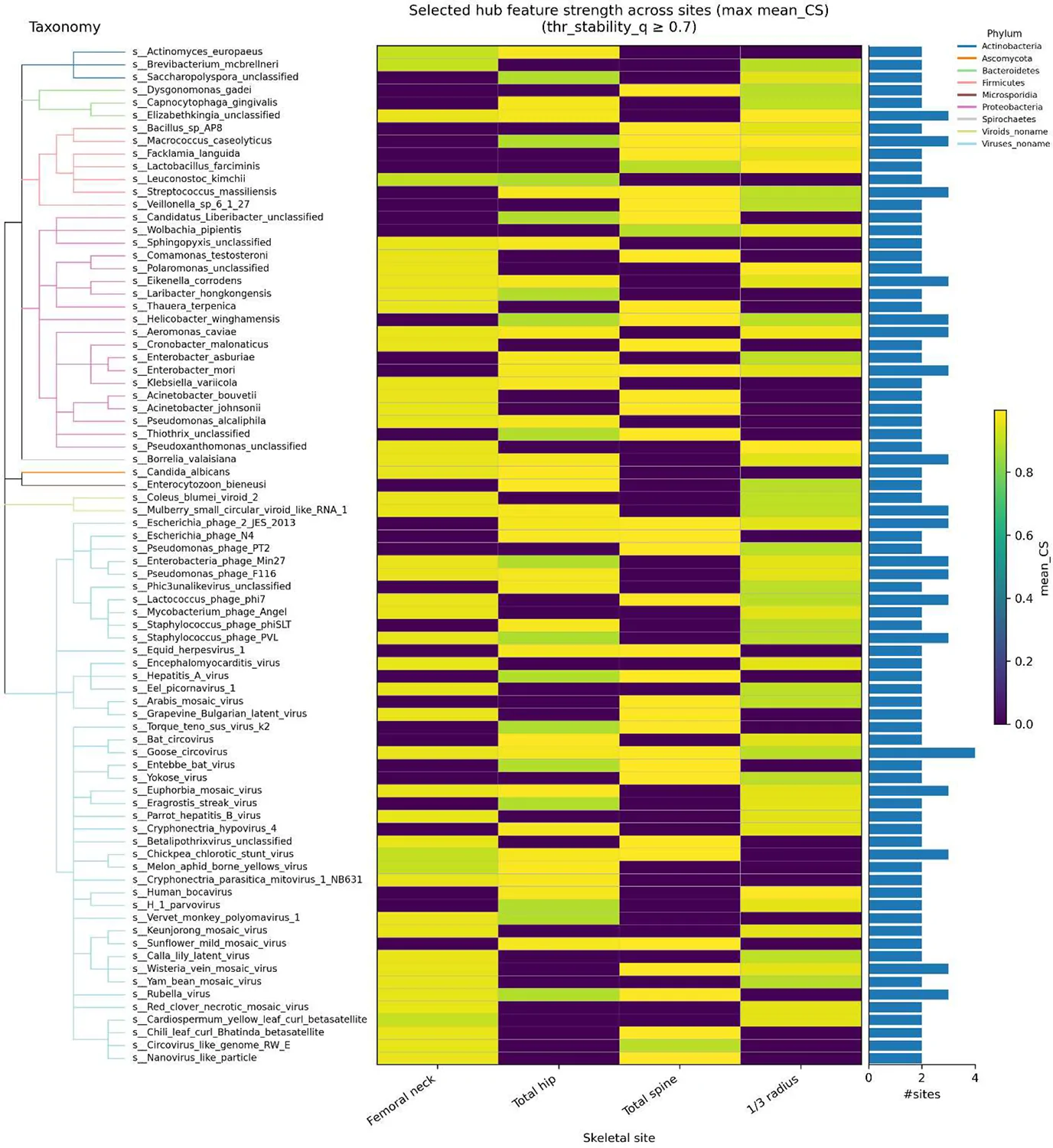
Cross-site summary of bootstrap-stable hub features. Heatmap showing the top-ranked bootstrap-stable hub features across skeletal sites in SAMECAT. Hub features were identified independently within each site using the feature-level clustering score (CS), which quantifies how strongly each microbial feature aligns with the attribution signature of its assigned module, together with bootstrap-based stability filtering. For cross-site visualization, features were ranked by the number of skeletal sites in which they were retained as hubs and, secondarily, by overall hub strength (sum of mean bootstrap CS) across sites. The heatmap displays the top 80 features, with cell color indicating mean bootstrap CS within each skeletal site. Rows are ordered by taxonomic similarity, shown by the phylogenetic tree at left, and the bar plot at right indicates the number of skeletal sites in which each feature was selected as a hub.

To summarize the microbial features most representative of attribution-driven modules, we next examined a ranked set of bootstrap-stable hub features across skeletal sites (see Methods for more details on hub feature identification). Hub features were first identified within each skeletal site using the feature-level clustering score (CS, which quantifies how strongly each species aligns with the signature attribution pattern of its assigned module, see Methods) and bootstrap stability filtering, and were then aggregated into a cross-site feature-by-site summary matrix. For visualization, we displayed the top 80 hub features, prioritizing features highlighted in more skeletal sites and, secondarily, features with greater overall hub strength (sum of mean bootstrap CS) across sites (Fig. 8).

Within this prioritized top-ranked set, several patterns were apparent. First, many displayed hub features showed cross-site recurrence, although their mean CS values varied across skeletal sites, indicating that some module-representative microbial signals were shared across multiple BMD phenotypes but differed in the strength of their within-site module representativeness. Second, these top-ranked hub features were concentrated within a limited number of phyla, suggesting that certain taxonomic groups may be disproportionately informative to the module-level predictive structure learned by the model.

Among the top-ranked bootstrap-stable hub features, viral taxa, Firmicutes, and Proteobacteria were the most frequently represented taxonomic groups. For Firmicutes, there is reasonably good literature support linking this group to bone phenotypes. In a human study of elderly adults, Firmicutes were enriched in controls and positively correlated with BMD and T-score, whereas low-BMD individuals showed a different phylum-level composition [64]. A human metagenomic plus metabolomic study in peri- and early post-menopausal women also reported that Firmicutes were positively associated with BMD [65]. For Proteobacteria, the literature is more mixed but still supportive of relevance. In a rat osteoporosis-related study, UV-associated improvement in bone density was accompanied by changes in Firmicutes and Proteobacteria [66]. A 2026 clinical recovery study in postmenopausal women linked elevated Proteobacteria to poorer orthopedic healing outcomes [67]. For the viral group, the gut virome is an important component of the gut ecosystem. Bacteriophages can shape bacterial community structure through lytic and lysogenic interactions, and the virome can also influence host immunity and inflammatory tone. In addition, recent reports suggest that phage-based therapies may hold promise as emerging therapeutic strategies for human metabolic diseases [68–70]. Because immune regulation, inflammation, and microbiome-mediated metabolism are already central mechanisms in the gut–bone axis [20, 71], it is reasonable to say that virome-related features may mark microbiome ecological states linked to bone-relevant metabolic or immune processes, although direct mechanistic roles cannot be inferred from the present data.

The prominent representation of viral taxa in the hub summary is particularly notable when viewed alongside the prevalence-filtering analysis. After removal of species-level features absent in more than 95% of BGI tuning set subjects, only four viral features were retained. As described above, restricting SAMECAT to the prevalence-filtered species reduced predictive performance relative to the full model across skeletal sites. Taken together, these observations further support the inference that rare microbial features, including virome-related taxa, may carry non-redundant information that contributes to the predictive signal structure learned by the model.

## Discussion

In this study, we developed SAMECAT, a structure-aware multi-view deep learning framework that integrates clinical covariates with species-level shotgun metagenomic relative-abundance profiles for host phenotype prediction. SAMECAT was motivated by the challenge that these two data views differ markedly in dimensionality, sparsity, and dependency structure, making effective integration more difficult than simple feature concatenation. By combining a taxonomy-aware encoder for microbiome data, an MLP encoder for clinical variables, and a prediction-calibrated fusion strategy guided by clustering-aware contrastive learning, SAMECAT achieved strong predictive performance on BMD across skeletal sites and retained its advantage under external evaluation using a metagenomic dataset processed through a different sequencing and bioinformatics workflow.

A central finding of this work is that SAMECAT’s advantage appears to arise from effective cross-view integration rather than model complexity alone. Conventional machine learning models remained competitive in the clinical-only setting, but they often failed to benefit from the addition of microbiome relative-abundance features. In contrast, SAMECAT showed clear improvement when microbiome and clinical information were jointly modeled, indicating that its representation-learning strategy is able to recover complementary predictive signal from high-dimensional metagenomic profiles. Comparisons with alternative integration strategies further support this inference: neither naive concatenation nor less-calibrated alignment approaches matched the performance of SAMECAT.

An important strength of SAMECAT is its robustness under cross-pipeline external evaluation. The external cohort differed from the development cohort not only in sequencing and bioinformatics workflow but also in demographic composition, creating a stringent test of generalizability. Despite these shifts, SAMECAT consistently outperformed machine learning baselines, suggesting that the learned multi-view representations capture predictor–outcome relationships that are more stable than those learned directly from the original input features by the machine-learning models. These results support the portability of the framework under realistic technical and demographic heterogeneity.

Beyond prediction, this study also introduces an interpretation workflow for extracting biologically meaningful structure from low-magnitude feature attributions. By combining EG with attribution-driven spectral clustering, bootstrap-guided stability analysis, pathway-level summarization, and hub-feature identification, we converted diffuse feature-level signals into reproducible module-, pathway-, and taxon-level summaries. The resulting patterns were biologically coherent yet site dependent. Pathway associations highlighted recurrent functional themes, including amino acid biosynthesis, nucleotide metabolism, central carbon metabolism, and one-carbon/cofactor metabolism, while also revealing substantial heterogeneity across skeletal sites. This heterogeneity might be partially attributed to the metabolic and biomechanical differences among femoral neck, total hip, total spine, and one-third radius.

The hub-feature analysis provided a complementary view of the learned predictive structure. Several hub taxa recurred across skeletal sites, whereas others were more site-restricted, suggesting a mixture of shared and site-specific microbiome-associated predictive programs. Notably, viral taxa remained prominent in the hub summary despite their limited prevalence, supporting the possibility that rare microbial features may carry non-redundant predictive information.

Several limitations should be acknowledged. First, although external evaluation is a major strength, broader replication across independent cohorts will be required to establish generalizability more fully. Second, the present analyses are predictive and interpretive rather than causal, such that the highlighted taxa and pathways should be regarded as prioritized candidates rather than confirmed mechanistic drivers; this motivates future work linking hub taxa and module-associated pathways to targeted experimental validation. Third, although the interpretation pipeline improves robustness, it remains dependent on methodological choices such as clustering parameters and stability thresholds, motivating future development of inference frameworks that more explicitly quantify attribution uncertainty [72, 73]. Beyond these limitations, extension of SAMECAT to richer multi-view settings represents a natural methodological direction for capturing additional complementary biological information. Finally, advancing the modality-specific subnetworks with more powerful representation-learning approaches might lead to further performance improvement.

In conclusion, our integrative framework for host phenotype prediction and interpretation supports more effective use of high-dimensional microbiome profiles in a multi-view setting while improving the transparency of learned predictive signals. Together, these advances contribute to the development of more trustworthy microbiome-based host phenotype modeling, in which learned signals are evaluated not only for accuracy but also for reproducibility, interpretability, and biological coherence.

## Methods

### Dataset

The Louisiana Osteoporosis Study (LOS) is an ongoing, population-based, cross-sectional cohort initiated in 2011 with the objective of establishing a large, well-characterized dataset to investigate genetic and environmental determinants of musculoskeletal and other complex traits, including osteoporosis, sarcopenia, and obesity. Adult participants (⩾ 18 years of age) were recruited from Baton Rouge, New Orleans, and surrounding regions in Louisiana. Detailed inclusion and exclusion criteria have been described in previous publications [74–77]. For the present analysis, we included 2,471 participants with complete genome-wide genotype data, shotgun metagenomic sequencing data, and valid bone mineral density (BMD) measurements at the femoral neck, total hip, total spine, and one-third radius. All participants provided written informed consent, and the study protocol was approved by the Institutional Review Board of Tulane University (No. 184088).

#### Covariates and BMD

Questionnaire survey was used to gather information such as demographics, anthropometry, digestive history, physical activity, and behavioral information. We included the following covariates in our analysis: age, gender, race, body mass index (BMI, calculated as weight (kg) / [height (m)]^2^), dairy product intake (milk [8-ounce cup/week], yogurt [oz/week], cheese [oz/week]), probiotic use (“yes” if the subject is currently taking or has taken probiotics in the past 12 months), exercise status (“yes” if the subject exercises at least one time per week), sun exposure status (“yes” if the subject receives at least 15 minutes of sun exposure per day), smoking status (“yes” if the subject has past history or current use of tobacco), alcohol drinking status (“yes” if the subject has past history or current use of alcohol). Femoral neck BMD, total hip BMD (combined BMD of the femoral neck, trochanter, and intertrochanteric region), total spine BMD (L1–L4), and one-third radius BMD were assessed for each participant using dual-energy X-ray absorptiometry (DXA) with a Hologic Discover-A DXA machine (Hologic Inc., Bedford, MA, United States). All scans were performed by trained and certified research personnel following standardized acquisition protocols. Instrument performance was monitored through daily calibration using a manufacturer-provided phantom, and measurement precision was evaluated based on the coefficient of variation derived from repeated scans. More information about BMD measurements and data quality control including the usual covariation for repeated measures can be found in our previous publications [75, 77–80]. We summarized the covariates and BMD information across all subjects in Table 1.

#### Global Ancestry Control

To control for confounding due to global genetic ancestry, we incorporated ancestry covariates derived from genome-wide Single Nucleotide Polymorphism (SNP) data into all numerical models. Global ancestry reflects broad-scale population structure arising from historical migration, admixture, and genetic drift, which manifests as systematic differences in allele frequencies across individuals [81, 82]. Such population structure can bias association analyses when ancestry is correlated with both the outcome and explanatory variables, including gut microbiome features [83, 84], leading to spurious or inflated associations—a phenomenon widely recognized as population stratification [81, 85]. Adjustment for ancestry is therefore critical in studies of complex traits to ensure valid inference. We summarized global ancestry using SNP-derived principal components (PCs), which capture the major axes of genetic variation attributable to population structure, and included these components as covariates to account for stratification effects.

Component-based ancestry adjustment has been extensively validated and is standard practice in genome-wide association studies and integrative analyses of human traits [82, 85]. Detailed procedures for whole-genome sequencing, variant calling, SNP quality control, linkage disequilibrium pruning, and principal component derivation are provided in the Supplementary Methods.

Specifically, self-reported race group was included to account for coarse ancestry structure, whereas genotype PC1 and PC2, selected based on the scree plot and cumulative genetic variance explained, were included to capture residual continuous ancestry variation. Higher-order PCs were not included to minimize redundancy and avoid over-adjustment.

#### Metagenomic Data Generation and Processing

Stool samples underwent shotgun metagenomic sequencing at two facilities, with distinct sequencing pipelines and downstream bioinformatic workflows applied at each site; 1,990 samples were processed at BGI Americas (Cambridge, MA) and 481 samples at LC Sciences (Houston, TX). Detailed procedures for sample collection, DNA extraction, library preparation, sequencing, and bioinformatic processing are provided in the Supplementary Methods.

### Modeling Framework

#### Overview

SAMECAT integrates shotgun metagenomic relative abundance profiles and clinical–demographic covariates through a structure-aware representation learning strategy in which each data view is first encoded using a neural subnetwork tailored to its intrinsic statistical and biological structure—an MLP for dense, low-dimensional clinical variables and a taxonomy-aware architecture for sparse, high-dimensional, hierarchically structured microbiome data. The framework emphasizes representation-level integration, leveraging a clustering-informed contrastive learning module to align and fuse modality-specific latent representations. Model training proceeds in two sequential phases, consisting of an unsupervised representation learning stage guided by deep clustering objectives, followed by a supervised prediction stage optimized for host phenotype prediction with contrastive learning regularization.

#### Taxonomy-Aware Microbiome Encoder

Metagenomic relative abundance profiles were encoded using a taxonomy-adaptive neural network architecture, adapted from MIOSTONE [9], which explicitly embeds microbial taxonomic hierarchy into the network structure [9]. In this architecture, microbial taxa are organized according to a predefined taxonomy tree, with each internal node representing a taxonomic group and each leaf node corresponding to a species-level feature, which is the input. Connections between layers mirror the subordinate relationships in the taxonomy. In our study, species-level taxonomic profiles generated by MetaPhlAn2 encode the complete taxonomic hierarchy within feature identifiers, spanning kingdom to species levels, and were therefore directly used to construct taxonomy-aware model architectures.

Let $x_{i}^{\left(m\right)}\in R^{p}$ denote the microbiome feature vector for subject i. The taxonomy-aware encoder maps $x_{i}^{\left(m\right)}$ into a latent representation $z_{i}^{\left(m\right)}$ through hierarchical aggregation and nonlinear transformations:

$$z_{i}^{\left(m\right)}=f_{\text{tax}}\left(x_{i}^{\left(m\right)}\right),$$

where $f_{\text{tax}}(\cdot )$ denotes the taxonomy-adaptive neural network. During model training, this deep neural network dynamically determines whether trait-relevant information is better captured at finer (e.g., species) or coarser (e.g., genus or family) taxonomic levels [9].

#### Clinical and Demographic Encoder

Clinical and demographic covariates, including age, sex, body mass index, and ancestry principal components, were encoded using a separate fully connected neural network:

$$z_{i}^{\left(c\right)}=f_{\text{clin}}\left(x_{i}^{\left(c\right)}\right),$$

where $x_{i}^{\left(c\right)}$ denotes the vector of non-microbiome covariates and $f_{\text{clin}}(\cdot )$ is an MLP producing a latent clinical representation.

#### Multi-View Representation Fusion

The microbiome and clinical representations were treated as two complementary views of each subject. Let ${\text{z}}_{i}^{\left(1\right)}={\text{z}}_{i}^{\left(m\right)}$ and ${\text{z}}_{i}^{\left(2\right)}={\text{z}}_{i}^{\left(c\right)}$. The fused representation was computed as a weighted linear combination:

$$z_{i}=\sum_{v=1}^{2}w_{v}z_{i}^{\left(v\right)},\text{subject to}\sum_{v=1}^{2}w_{v}=1,w_{v}>0,$$

where the fusion weights $w_{v}$ are learnable parameters obtained via a SoftMax transformation. This formulation allows the model to adaptively prioritize microbiome or clinical information based on their relevance to the learning objective.

#### Phase I — Deep Divergence-based Clustering

In the first training phase, the model was optimized using a deep divergence-based clustering (DDC) [34, 35] objective. Let $z_{i}\in R^{d}$ denote the fused latent representation of subject i. Let $h_{i}\in R^{l}$ denote the hidden representation obtained by passing the fused representation $z_{i}$ through a fully connected layer. A kernel matrix $\kappa \in R^{n\times n}$ is defined as $\kappa_{ab}=\text{exp}\left(-{\left\|h_{a}-h_{b}\right\|}^{2}/\left(2\sigma^{2}\right)\right)$, which measures similarity between subjects. A clustering head maps $h_{i}$ to a soft cluster assignment vector

$$\alpha_{i}=\left(\alpha_{i1},\ldots ,\alpha_{iK}\right)\in R^{K},$$

where K is the number of latent clusters and $\sum_{k=1}^{K}\alpha_{ik}=1$.

##### 1. Cluster Compactness and Separability Loss

The first loss term encourages compact clusters while promoting separation between different clusters:

ℒ1=∑i<jK2−1∑a,bαaiκabαbj∑a,bαaiκabαbi∑abαajκabαbj

Here, $\alpha_{ai}$ denotes the soft assignment of sample a to cluster i. This term penalizes overlap between different samples’ cluster assignment distributions when their associated clusters are different.

##### 2. Orthogonality Loss on Cluster Assignments

To prevent degenerate solutions in which samples collapse into identical cluster assignments, an orthogonality constraint is imposed:

ℒ2=n2−1∑i<jαi⊤αj

This term encourages cluster assignment vectors from different samples to be distinct, promoting diversity across clusters.

##### 3. Simplex Corner Regularization

The third loss term encourages soft assignments to concentrate near the vertices of the probability simplex. Let ${\text{e}}_{j}\in R^{K}$ denote the j-th canonical basis vector (i.e., a one-hot vector with a 1 in position j and 0 elsewhere). Define

$$m_{ij}=\text{exp}\left(-{\left\|\alpha_{i}-e_{j}\right\|}^{2}\right),$$

which measures the proximity of sample i’s assignment vector to the j-th simplex corner.

The simplex regularization loss is then defined as:

ℒ3=∑i<jK2−1∑a,bmaiκabmbj∑a,bmaiκabmbi∑a,bmajκabmbj

This term promotes confident (near one-hot) cluster assignments while preserving cluster separation.

#### Total Clustering Objective

The overall deep divergence-based clustering loss is defined as:

$${ℒ}_{\text{cluster}}={ℒ}_{1}+{ℒ}_{2}+{ℒ}_{3}.$$

#### Phase II: Host Phenotype Prediction with Contrastive Learning Regularization

In the second phase, the model was fine-tuned for supervised host phenotype (BMD in this study) prediction. A regression head was applied to the fused representation:

$${\overset{ˆ}{y}}_{i}=g\left(z_{i}\right),$$

where ${\overset{ˆ}{y}}_{i}$ denotes predicted phenotype and g(⋅) is a fully connected MLP trained using a regression loss (e.g., mean squared error).

To regularize multi-view integration, we incorporated a contrastive learning (CL) objective that selectively aligns representations across views at the sample level, rather than enforcing global distribution alignment [35]. For views v≠u, cosine similarity was computed as:

$$s_{ij}^{\left(vu\right)}=\frac{z_{i}^{(v)⊤}z_{j}^{\left(u\right)}}{\left\|z_{i}^{\left(v\right)}\right\|\cdot \left\|z_{j}^{\left(u\right)}\right\|}$$

The contrastive loss was defined as a generalized **NT-Xent** (the normalized temperature-scaled cross entropy) objective [28]:

$${ℒ}_{\text{CL}}=\frac{1}{nV(V-1)}\sum_{i=1}^{n}\sum_{v\neq u}\left[-\text{log}\frac{\text{exp}\left(s_{ii}^{\left(vu\right)}/\tau \right)}{\sum_{s^{\prime }\in {𝒩}_{i}}\text{exp}\left(s^{\prime }/\tau \right)}\right],$$

where τ is a temperature parameter and ${𝒩}_{i}$ denotes negative pairs drawn from samples assigned to different clusters.

The final training objective was:

$$ℒ={ℒ}_{\text{pred}}+\lambda \cdot \text{min}\left(w_{1},w_{2}\right){ℒ}_{\text{CL}},$$

ensuring that contrastive alignment is adaptively applied and does not override view prioritization [35].

### Model Training, Data Partitioning, and Evaluation Design

#### Internal Evaluation Design and Model Benchmarking

Internal evaluation was performed using the BGI-derived dataset to support model framework development, hyperparameter selection, and comparative benchmarking across modeling approaches. The BGI data were randomly partitioned into a tuning set and a testing set, with the testing set held out entirely from model training and hyperparameter optimization to prevent information leakage.

Within the tuning set, samples were further divided into training and validation subsets. For each modeling approach, hyperparameter tuning was performed by fitting models under different candidate hyperparameter configurations on the training subset and evaluating the resulting models on the validation subset. The configuration yielding the best validation performance was then selected, after which the model was retrained on the full tuning set and evaluated on the held-out testing set.

To assess robustness to training/validation partitioning, we generated 10 random training/validation partitions within the BGI tuning set. For each method, hyperparameter tuning, model retraining, and evaluation on the held-out testing set were performed independently for each partition, yielding 10 testing-set performance estimates per method.

#### External Validation Design

External validation was conducted using the LC-derived dataset to evaluate the robustness and generalizability of the developed models across metagenomic sequencing and bioinformatics processing pipelines.

For this analysis, models trained on the BGI tuning set using best hyperparameter configurations selected from the corresponding training/validation partition during internal evaluation were directly applied to the LC-derived dataset without additional retraining.

This design ensures that all methods were evaluated under identical training conditions, with the LC-derived dataset serving solely as an external test set for assessing cross-pipeline model robustness.

#### Model Selection for Interpretation

For downstream results interpretation, Expected Gradients (EG) attribution matrices were computed for samples in the BGI-derived testing set, using the BGI tuning set as the reference distribution [25]. For each skeletal site, a representative trained model instance was selected based on internal evaluation performance and used for site-specific attribution and downstream interpretability analyses.

### Model Interpretation and Result Attribution Analysis

To interpret predictive relationships learned by the proposed deep learning framework, we developed a multi-stage model interpretation pipeline that integrates gradient-based feature attribution, attribution-driven module discovery, stability-guided hyperparameter selection, functional pathway summarization, and hub feature identification. In this study, the interpretation pipeline was applied independently to models trained for femoral neck, total hip, total spine, and one-third radius BMD prediction, enabling both site-specific interpretation and cross-site comparison.

#### Expected Gradient–Based Feature Attribution

Feature-level contributions to model predictions were quantified using EG, a gradient-based attribution method that extends Integrated Gradients by averaging attribution paths over a reference distribution, thereby improving stability with respect to baseline selection [25, 86].

Let f(x) denote the trained model’s prediction for input x, and let $x_{j}$ denote the j-th microbiome feature. The EG attribution for feature j is defined as:

$${\text{EG}}_{j}\left(\text{x}\right)=E_{{\text{x}}^{\prime }\sim 𝒟}\left[\left(x_{j}-x_{j}^{\prime }\right)\int_{0}^{1}\frac{\partial f\left({\text{x}}^{\prime }+\alpha \left(\text{x}-{\text{x}}^{\prime }\right)\right)}{\partial x_{j}}d\alpha \right],$$

where ${\text{x}}^{\prime }$ is sampled from an empirical reference distribution 𝒟. In this study, the reference distribution 𝒟 was defined as the empirical distribution of observed microbiome feature vectors derived from the BGI tuning set, from which baseline samples were randomly drawn for EG computation. Here, α∈[0,1] parameterizes a straight-line interpolation between the baseline input $x^{\prime }$ and the observed testing set input x.

For each skeletal site, EG attributions were computed at the subject level and assembled into a subject-by-feature attribution matrix, which served as the basis for downstream module discovery and functional/taxonomic interpretation.

#### Attribution-Driven Spectral Clustering of Microbial Features

To identify groups of microbial features exhibiting coherent predictive contribution patterns, spectral clustering was applied to the EG attribution matrix [87, 88]. Prior to clustering, Expected Gradient values were transformed using a scale-normalized signed logarithmic transformation:

$${\tilde{E}}_{ij}=\text{sign}\left(E_{ij}\right)\cdot \text{log}\left(1+\frac{\left|E_{ij}\right|}{\epsilon }\right),\epsilon =\text{median}\left\{\left|E_{ij}\right|:\left|E_{ij}\right|>0\right\}.$$

Normalization by the median absolute non-zero attribution magnitude provides a robust, data-adaptive scaling factor whose estimation is less sensitive to extreme values, while preserving relative contribution patterns across features and subjects.

Feature–feature similarity was computed using cosine similarity between EG attribution profiles across subjects. A k-nearest-neighbor affinity graph was constructed, and spectral clustering was performed on the normalized graph Laplacian to derive attribution-driven microbial modules. Details on hyperparameter tuning and stability assessment for spectral clustering can be found in Supplementary Methods.

#### Pathway-Level Interpretation and Pathway Relevance Score (PaRS)

Pathway abundance was defined at the subject level based on functional profiling of shotgun metagenomic data [40] and was subsequently aggregated at the module level by restricting pathway signals to species assigned to each attribution-driven module. The resulting module-level pathway abundance vectors across subjects were used in downstream correlation and bootstrap stability analyses. Pathway coverage [40] was aggregated in an analogous manner and incorporated into the Pathway Relevance Score (PaRS) to emphasize pathways that were both quantitatively abundant and structurally well represented within each module. Within each module, pathway abundance was aggregated across contributing species by summation, whereas pathway coverage was aggregated by taking the mean, reflecting total functional contribution and average pathway completeness, respectively. To translate attribution-driven microbial modules into functional insights, we performed module-wise pathway analysis by integrating pathway abundance, pathway coverage, and attribution–trait concordance.

For each module–pathway pair, three quantities were computed:

- the Spearman correlation coefficient (ρ) between the module-level attribution centroid and pathway abundance across subjects;
- mean aggregated pathway coverage across subjects; and
- a bootstrap-based stability measure (described below).

These components were combined into a Pathway Relevance Score (PaRS):

$$\text{PaRS}=|\rho |\times \text{log}(1+\text{cov}\overset{-}{\text{er}}\text{age})\times \text{stability}.$$

The multiplicative form of PaRS reflects a conjunctive relevance criterion, whereby pathways are prioritized only if they simultaneously exhibit strong attribution concordance, sufficient functional presence, and robustness under resampling. This formulation prevents any single component from dominating the score and naturally downweighs pathways that fail to satisfy one or more interpretability criteria.

To restrict interpretation to pathways exhibiting non-trivial association with module-level attribution signals and sufficient representation across subjects, we applied tuned screening thresholds, candidate pathways were required to satisfy minimum coverage and prevalence criteria based on module-level aggregated pathway coverage, as well as a minimum absolute association magnitude (|Spearman ρ|) threshold. Threshold selection was performed independently for each skeletal site using grid search, prioritizing high bootstrap reproducibility among top-ranked (top 10) module–pathway pairs while retaining a meaningful number of associations for interpretation (see Supplementary Methods). For each module–site combination, Spearman correlation p-values across retained pathways were adjusted for multiple comparisons using the Benjamini–Hochberg false discovery rate (FDR) procedure [89, 90], and pathways with q-value > 0.10 were excluded from downstream interpretation.

#### Bootstrap Stability Assessment in Pathway Analysis

To assess the robustness of module–pathway relationships and reduce sensitivity to sampling variability, we incorporated a bootstrap-based stability assessment into the pathway-level interpretation pipeline.

For each module–pathway pair, subjects were resampled with replacement for B=200 bootstrap iterations. In each resample, the Spearman correlation between the module-level EG signal and pathway abundance was recomputed. A resample was considered consistent if the direction of association matched that observed in the original dataset and Spearman correlation p-value is less than 0.05.

The stability score was defined as the proportion of bootstrap resamples satisfying this consistency criterion:

$$\text{stability}=\frac{1}{B}\sum_{b=1}^{B}I(\text{consistent association in resample}\;b),$$

where I(⋅) denotes the indicator function.

### Feature-Level Clustering Score

To quantify how well individual microbial features represent the signature attribution pattern of their assigned module, we defined a feature-level Clustering Score (CS). Let $E^{\left(t\right)}$ denote the transformed EG attribution matrix after application of the scale-normalized signed logarithmic transformation, where $E_{ij}^{\left(t\right)}$ is the transformed attribution of feature j in subject i. For a module m containing feature set $M_{m}$, the clustering score for feature $j\in M_{m}$ was defined as the Pearson correlation between the subject-level attribution profile of feature j and the leave-one-out mean attribution profile of the remaining features in the same module:

$${\text{CS}}_{j}=\text{corr}\left(E_{\cdot j}^{\left(t\right)},\frac{1}{\left|M_{m}\right|-1}\sum_{k\in M_{m},k\neq j}E_{\cdot k}^{\left(t\right)}\right).$$

This leave-one-out formulation avoids inflating coherence by correlating a feature with a centroid that includes itself. Higher CS values indicate stronger alignment of a feature with the signature attribution pattern of its assigned module.

#### Bootstrap-stable hub feature identification

To identify microbial features that robustly represented attribution-driven modules, we applied a bootstrap-based hub selection procedure within each skeletal site. Subjects were resampled with replacement for 300 bootstrap iterations, and feature-level CS were recomputed from the transformed EG attribution matrix while preserving the module assignments. Within each bootstrap replicate and module, a feature was counted as a hub “hit” if its CS was at or above the module-specific 90th percentile of the defined (not *NA*) CS values in that module. For each feature, we calculated the proportion of bootstrap replicates in which it satisfied this module-relative threshold (threshold stability), the fraction of replicates in which CS was defined, and the mean CS across bootstrap replicates. Features were retained as stable hub features if defined in at least 90% of bootstrap replicates, with threshold stability ≥ 0.70 and mean CS ≥ 0.50. For cross-site visualization, retained features were summarized in a feature-by-site matrix, and heatmap values were defined as the mean bootstrap CS observed for each feature within each skeletal site. Hub identification was restricted to positive CS values, as the objective was to identify features aligned with the signature within-module attribution pattern. Negative CS values indicate anti-alignment with the module signature and were therefore not considered hub-like.

### Statistical Analysis

Predictive performance was evaluated across repeated BGI tuning data partitions. For RMSE, paired comparisons between models were performed using paired t-tests. For $R^{2}$, paired comparisons were performed using the Wilcoxon signed-rank test. Here, $R^{2}$ was calculated using the squared Pearson correlation between predicted and observed BMD values, such that it quantified the strength of linear concordance between predictions and observed outcomes while emphasizing consistency in their overall trend across subjects. For the SAMECAT model using metagenomic relative abundance alone, certain BGI tuning data partitions at each skeletal site led to identical predictions for the BGI testing-set samples. As a result, valid paired differences in $R^{2}$ were too sparse in some comparisons, leading to undefined (*NA*) results for the Wilcoxon signed-rank test.

## Supplementary Material

1

Supplementary Files

This is a list of supplementary files associated with this preprint. Click to download.

- SupplementaryMethods.docx

## Figures and Tables

**Table 1.** Demographic, clinical, lifestyle, and bone mineral density characteristics of the samples stratified by sequencing cohort and sex.

	BGI		LC
	Male	Female	Male
Total Count	686	1304	481
Age	44.46 ± 13.45	49.13 ± 14.35	36.39 ± 8.29
BMI (kg/m^2^)	26.79 ± 4.73	25.89 ± 5.20	27.17 ± 5.16
Femoral neck BMD (g/cm^2^)	0.852 ± 0.129	0.767 ± 0.122	0.873 ± 0.136
Hip total BMD (g/cm^2^)	0.992 ± 0.126	0.903 ± 0.121	0.991 ± 0.127
Spine total BMD (g/cm^2^)	1.033 ± 0.136	0.991 ± 0.140	1.018 ± 0.134
1/3 radius BMD (g/cm^2^)	0.795 ± 0.063	0.690 ± 0.062	0.795 ± 0.057
Milk consumption (8-ounce cups/week)	5.89 ± 7.45 (20.4% missing)	4.79 ± 6.00 (6.9% missing)	7.28 ± 10.55
Yogurt consumption (oz/week)	6.14 ± 12.35 (9.8% missing)	11.74 ± 16.05 (5.8% missing)	8.2 ± 17.8
Cheese consumption (oz/week)	6.42 ± 8.10 (16.5% missing)	5.49 ± 5.78 (8.0% missing)	8.3 ± 12.49
Probiotic use (No / Yes)	600 (87.5%) / 65 (9.5%) (3.1% missing)	810 (62.1%) / 471 (36.1%) (1.8% missing)	451 (93.8%) / 30 (6.2%)
Caucasian / African American / Asian	362 (52.8%) / 301 (43.9%) / 23 (3.4%)	1027 (78.8%) / 225 (17.3%) / 52 (4.0%)	331 (68.8%) / 150 (31.2%) / none
Exercise ≥1×/week (No / Yes)	157 (22.9%) / 529 (77.1%)	325 (24.9%) / 979 (75.1%)	120 (24.9%) / 361 (75.1%)
Sun exposure ≥15 min/day (No / Yes)	97 (14.1%) / 589 (85.9%)	237 (18.2%) / 1067 (81.8%)	61 (12.7%) / 420 (87.3%)
Smoke (No / Yes)	255 (37.2%) / 431 (62.8%)	837 (64.2%) / 467 (35.8%)	129 (26.8%) / 352 (73.2%)
Drink alcohol (No / Yes)	179 (26.1%) / 480 (70.0%) (3.9% missing)	230 (17.6%) / 1066 (81.7%) (0.6% missing)	172 (35.8%) / 309 (64.2%)

Missing data were imputed using R package MICE [26]. To prevent information leakage, imputation was performed independently within the BGI tuning and testing sets (see Methods for more details on data partition).

## Data Availability

The Louisiana Osteoporosis Study (LOS) datasets underlying this study are available from the principal investigator, H.W.D. (hdeng2@tulane.edu), upon reasonable request for academic research purposes and subject to Institutional Review Board approval and a data use agreement. The LOS whole-genome sequencing (WGS) data are also in the process of being deposited in the Aging Research Biobank (https://agingresearchbiobank.nia.nih.gov/), through which they will be made available to qualified researchers upon application and approval. The LOS metagenomic sequencing (MGS) data can be found in “Sequence Read Archive” (https://www.ncbi.nlm.nih.gov/sra, accession No. PRJNA1015234 and PRJNA1015228).

## Code Availability

The SAMECAT repository (https://github.com/jianglindong93/SAMECAT) provides scripts for model training, internal and external evaluation, attribution computation, attribution-driven module discovery, pathway association analysis, and hub-feature summarization.
