# Supplementary material for: Robust and Interpretable Metagenomic Modeling Through Structure-Aware Multi-View Learning and Attribution-Guided Biological Insight
Source: Res Sq. 2026 Aug 5:rs.3.rs-9956795. Preprint. [Version 1] doi: 10.21203/rs.3.rs-9956795/v1 (PMC13484852; doi:10.21203/rs.3.rs-9956795/v1)
Supplement: Supplementary file 1 [file NIHPPRS9956795V1-supplement-1.pdf]

## Supplementary Table and Figures

**Supplementary Table S1. Summarization of species-level metagenomic data**

| | Number of species-level features before prevalence filtering | Number of species-level features with only zero relative abundance in tuning set samples | Number of species-level features after prevalence filtering | Number of species in common |
|---|---|---|---|---|
| BGI | 1269 | 43 | 266 | 697 |
| LC | 24721 | --- | --- | |

Species-level microbial features with zero relative abundance across all samples in the BGI tuning set were excluded. After this filtering step, 1,226 species-level microbial features were retained for model development.

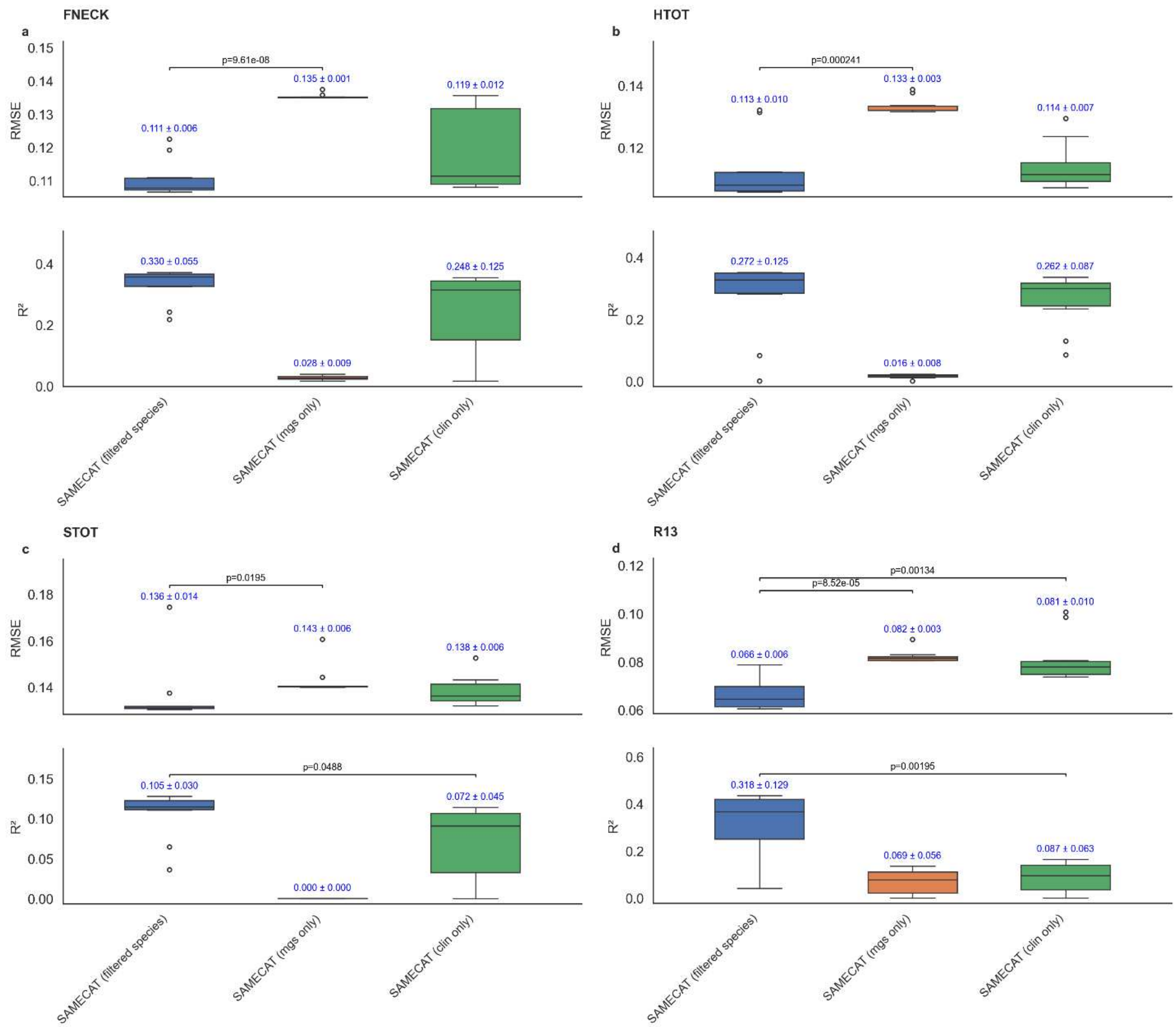


**Supplementary Figure S1. Performance of SAMECAT with prevalence-filtered microbiome input across skeletal sites.** Performance of SAMECAT was evaluated using multi-view input with prevalence-filtered microbial species set (species prevalence ≥5% in the BGI tuning set), microbiome-only input (all species retained), and clinical-only input for **(a)** femoral neck (FNECK), **(b)** total hip (HTOT), **(c)** total spine (STOT), and **(d)** one-third radius (R13) bone mineral density (BMD). Held-out test performance is shown as RMSE (top) and $R^2$ (bottom), with blue text denoting mean ± SD. Boxplots summarize model performance across 10 random training/validation partitions of the BGI tuning set used for hyperparameter tuning. For each partition, the selected

optimal hyperparameters were used to retrain the model on the full tuning set and evaluate performance on the held-out BGI testing set. P values for significant pairwise comparisons ($p < 0.05$) are shown above the boxplots; paired t-tests were used for RMSE and Wilcoxon signed-rank tests were used for $R^2$.

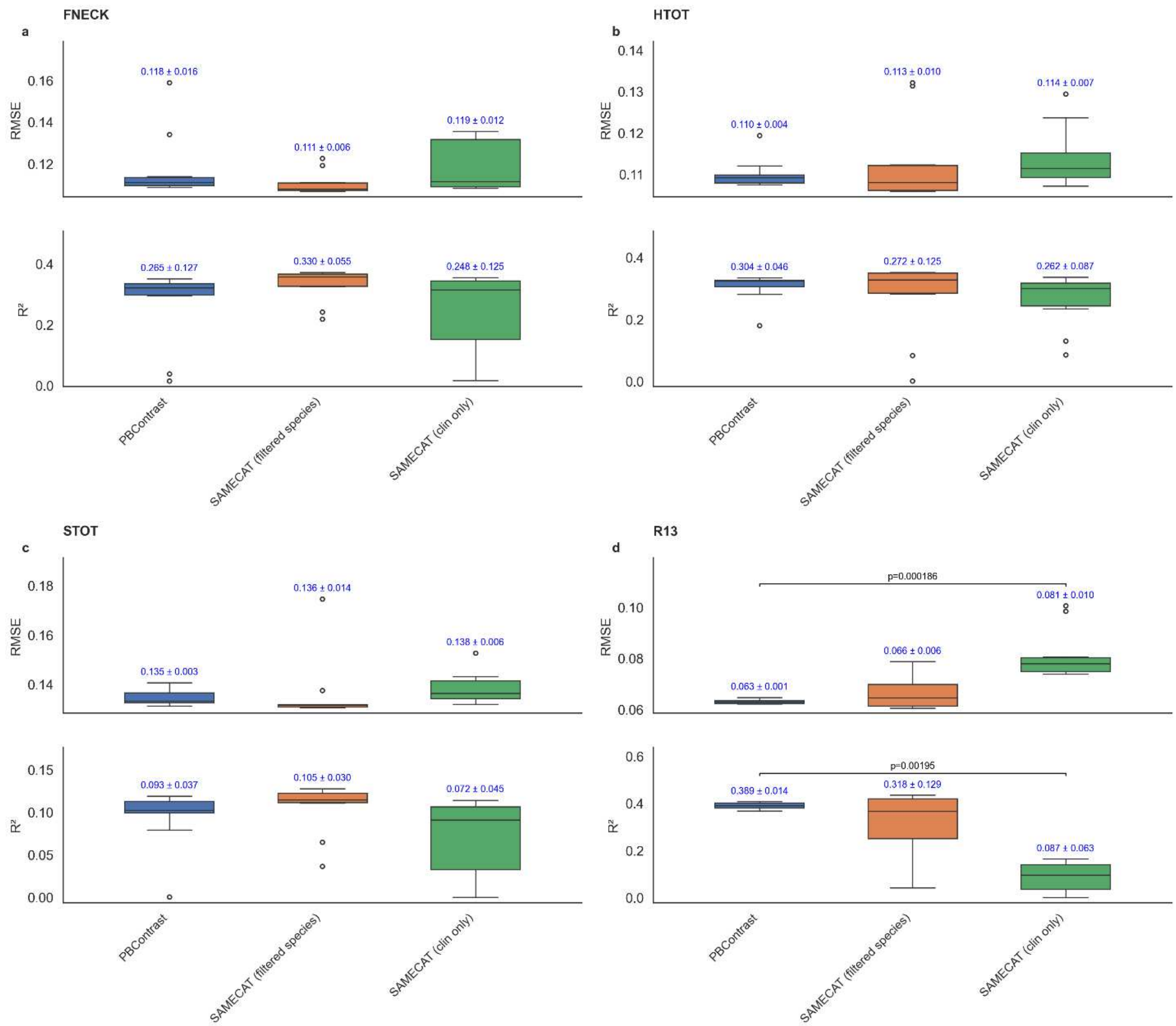


**Supplementary Figure S2. SAMECAT and PBContrast show comparable performance under matched prevalence-filtered input.** SAMECAT and PBContrast were compared in a controlled setting where both models were trained using the same prevalence-filtered microbiome species set, enabling assessment of representation-learning method independent of feature availability across **(a)** femoral neck (FNECK), **(b)** total hip (HTOT), **(c)** total spine (STOT), and **(d)** one-third radius (R13) BMD. Performance on held-out test data is shown across skeletal sites using RMSE (top) and $R^2$ (bottom), with blue text denoting mean ± SD. Boxplots summarize model performance across 10 random training/validation partitions of the BGI tuning set used for hyperparameter tuning. For each

partition, the selected optimal hyperparameters were used to retrain the model on the full tuning set and evaluate performance on the held-out BGI testing set. P values for significant pairwise comparisons ($p < 0.05$) are shown above the boxplots; paired t-tests were used for RMSE and Wilcoxon signed-rank tests were used for $R^2$. Performance for SAMECAT with only clinical variables as input is also presented for comparison.

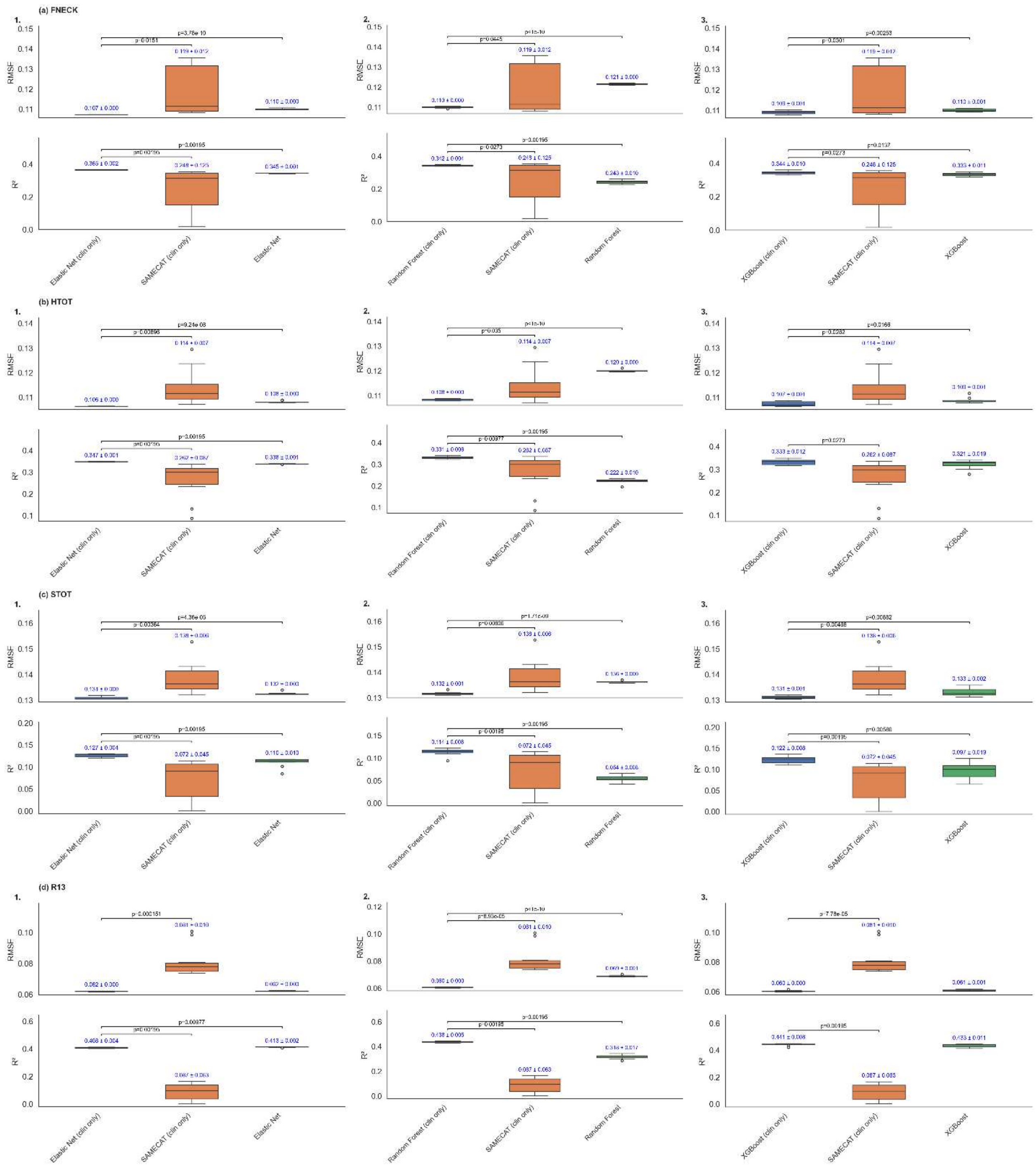


**Supplementary Figure S3. Machine-learning baselines outperform the clinical-only SAMECAT variant.** Performance under a clinical-only input setting was evaluated for SAMECAT clinical-only (Multi-Layer Perceptron), Elastic Net, Random Forest, and XGBoost across **(a)** femoral neck

(FNECK), **(b)** total hip (HTOT), **(c)** total spine (STOT), and **(d)** one-third radius (R13) bone mineral density (BMD). Held-out test performance is shown as RMSE (top) and $R^2$ (bottom), with blue text denoting mean ± SD. Boxplots summarize model performance across 10 random training/validation partitions of the BGI tuning set used for hyperparameter tuning. For each partition, the selected optimal hyperparameters were used to retrain the model on the full tuning set and evaluate performance on the held-out BGI testing set. P values for significant pairwise comparisons ($p < 0.05$) are shown above the boxplots; paired t-tests were used for RMSE and Wilcoxon signed-rank tests were used for $R^2$. Performance for machine learning baselines with both metagenomic relative abundance and clinical variables as input is also presented for comparison.

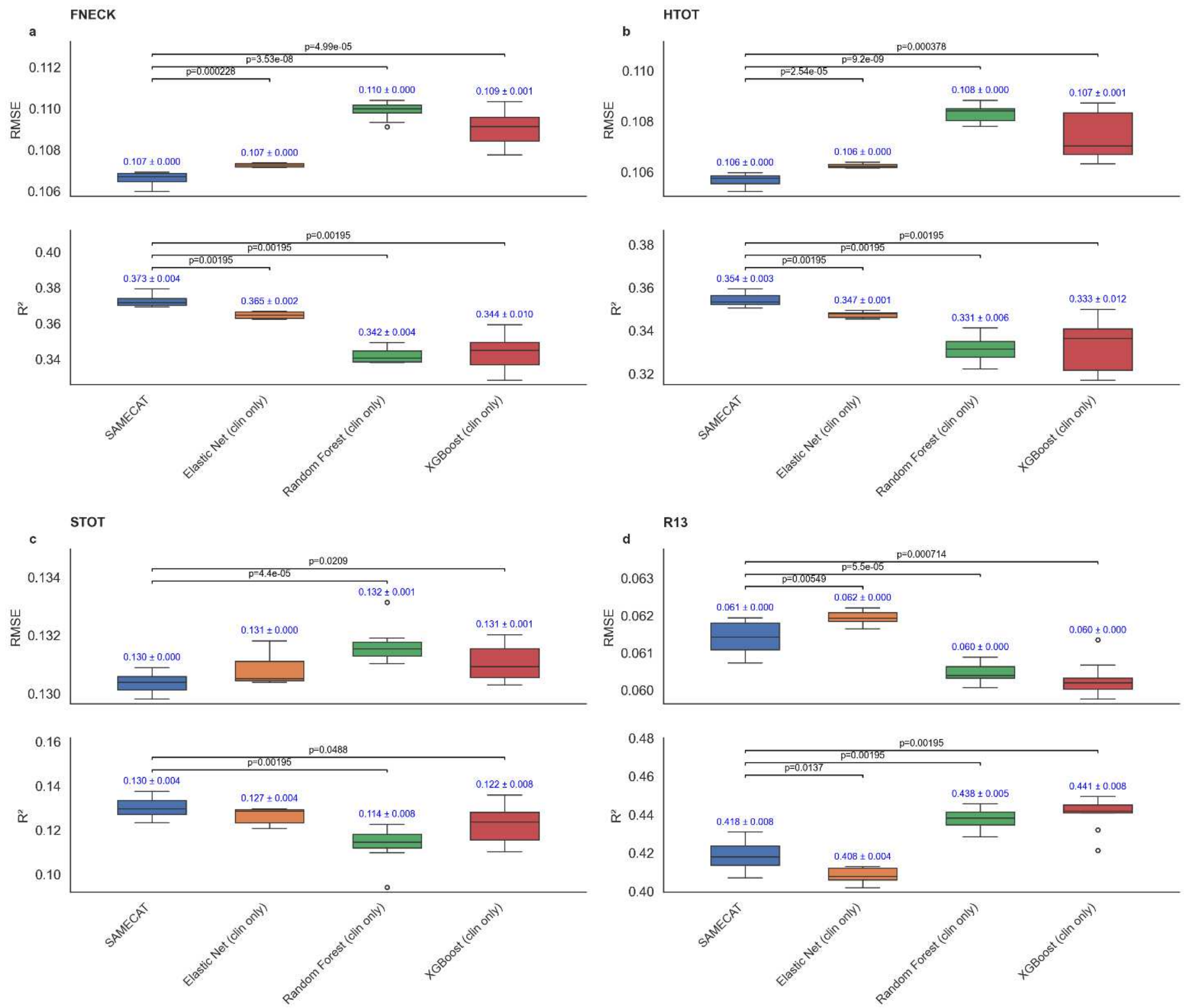


**Supplementary Figure S4. Full SAMECAT outperforms clinical-only machine-learning baselines at most skeletal sites.** Predictive performance of the full SAMECAT model (clinical + microbiome inputs) was compared with Elastic Net, Random Forest, and XGBoost trained using clinical covariates alone for **(a)** femoral neck (FNECK), **(b)** total hip (HTOT), **(c)** total spine (STOT), and **(d)** one-third radius (R13) BMD. Held-out test performance is shown as RMSE (top) and $R^2$ (bottom), with blue text denoting mean ± SD. Boxplots summarize model performance across 10 random training/validation partitions of the BGI tuning set used for hyperparameter tuning. For each partition, the selected optimal hyperparameters were used to retrain the model on the full tuning

set and evaluate performance on the held-out BGI testing set. P values for significant pairwise comparisons ($p < 0.05$) are shown above the boxplots; paired t-tests were used for RMSE and Wilcoxon signed-rank tests were used for $R^2$.

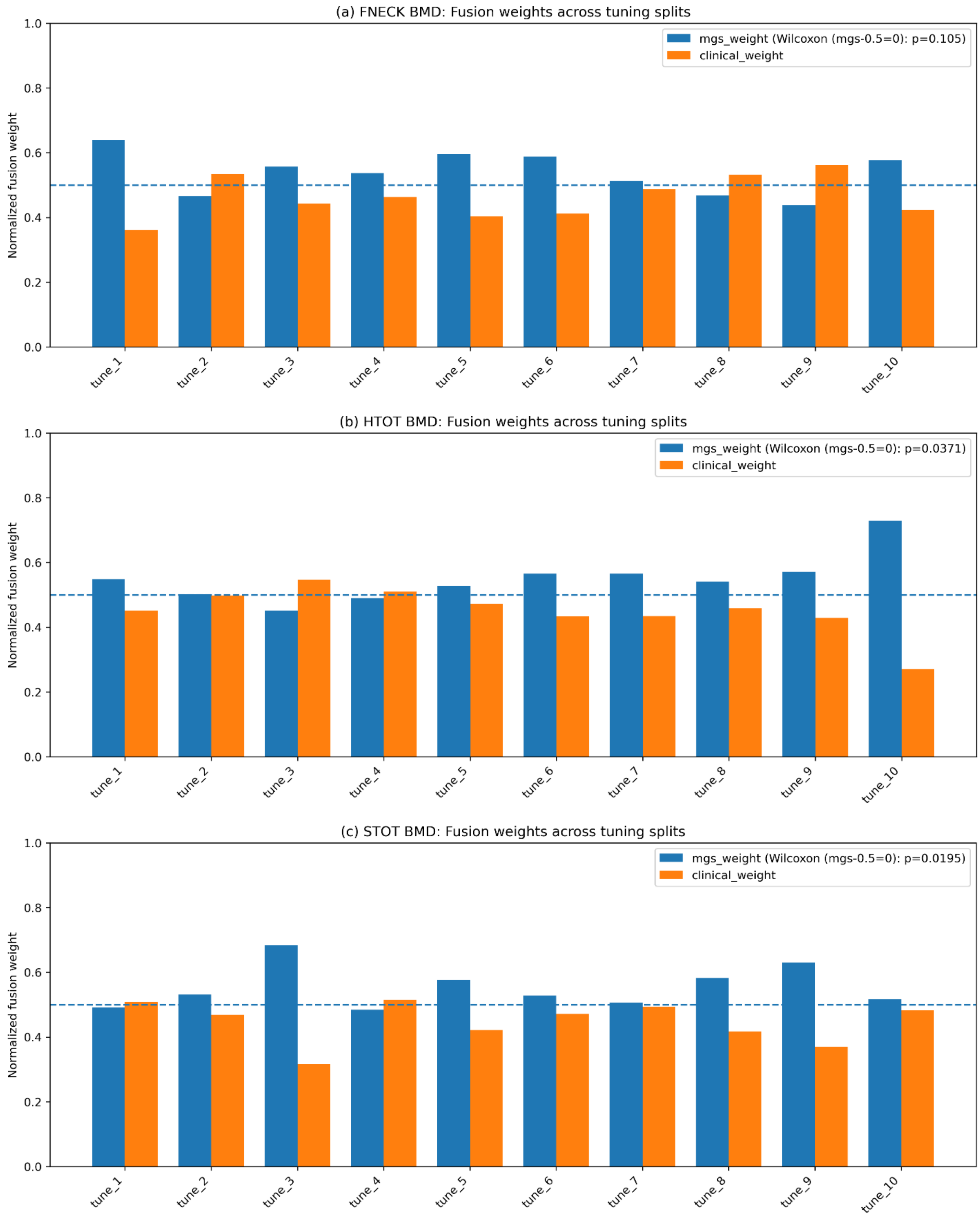

(a) FNECK BMD: Fusion weights across tuning splits
mgs_weight (Wilcoxon (mgs-0.5=0): p=0.105)
clinical_weight
Normalized fusion weight
0.0
0.2
0.4
0.6
0.8
1.0
tune_1
tune_2
tune_3
tune_4
tune_5
tune_6
tune_7
tune_8
tune_9
tune_10
(b) HTOT BMD: Fusion weights across tuning splits
mgs_weight (Wilcoxon (mgs-0.5=0): p=0.0371)
clinical_weight
Normalized fusion weight
(c) STOT BMD: Fusion weights across tuning splits
mgs_weight (Wilcoxon (mgs-0.5=0): p=0.0195)
clinical_weight
Normalized fusion weight

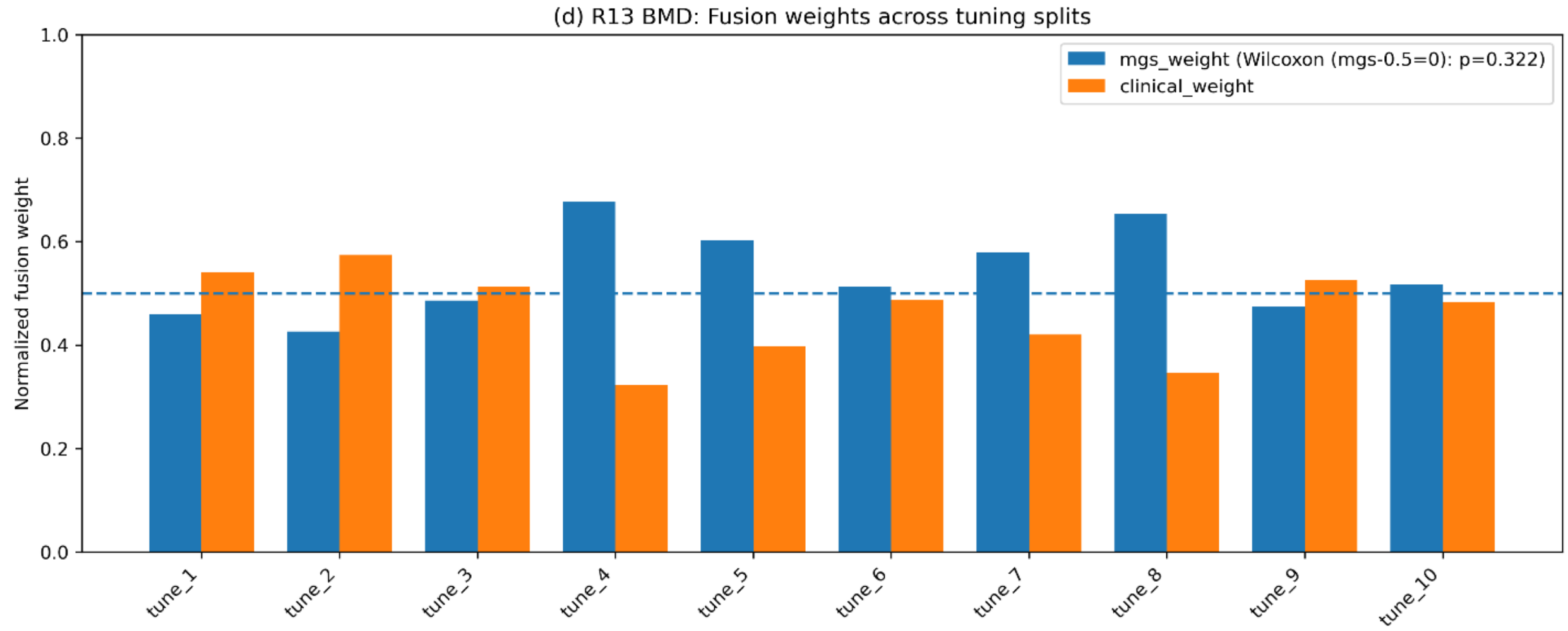


**Supplementary Figure S5. Relative view-specific contributions inferred from SAMECAT fusion weights.** SoftMax-normalized fusion weights are shown for microbiome- and clinical-derived latent representations across 10 SAMECAT models trained on the BGI tuning set using the best hyperparameter configurations selected from the 10 training/validation data partitions, for predicting **(a)** femoral neck (FNECK), **(b)** total hip (HTOT), **(c)** total spine (STOT), and **(d)** one-third radius (R13) BMD. The dashed horizontal line marks 0.5, indicating equal contribution of the two modalities. Wilcoxon signed-rank tests were used to test whether microbiome fusion weights differed from 0.5 across partitions. Microbiome-derived fusion weights were significantly greater than 0.5 for total hip and total spine, while femoral neck and one-third radius showed non-significant trends in the same direction.

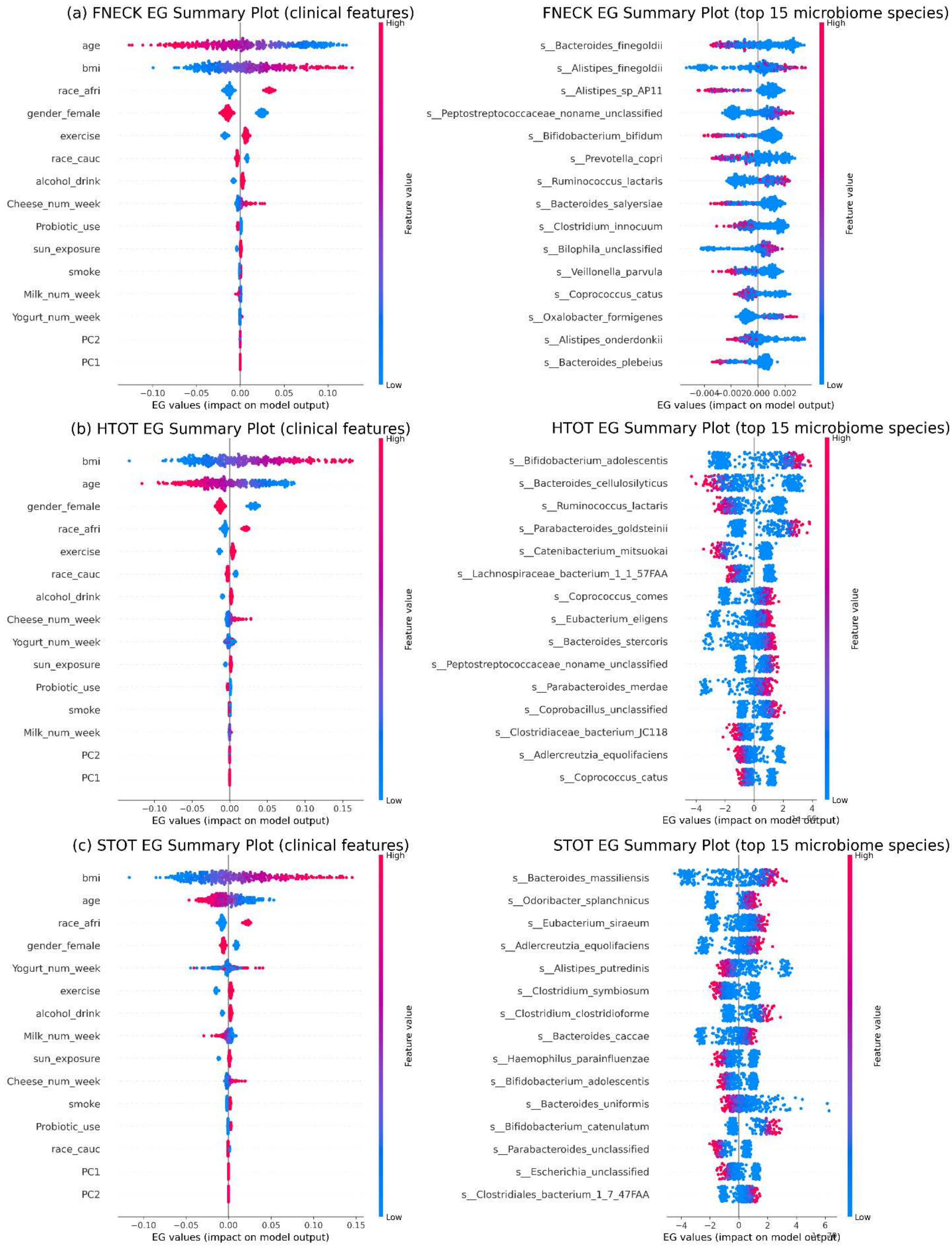
(a) FNECK EG Summary Plot (clinical features)
age
bmi
race_afri
gender_female
exercise
race_cauc
alcohol_drink
Cheese_num_week
Probiotic_use
sun_exposure
smoke
Milk_num_week
Yogurt_num_week
PC2
PC1
−0.10 −0.05 0.00 0.05 0.10
EG values (impact on model output)
High
Feature value
Low
FNECK EG Summary Plot (top 15 microbiome species)
s__Bacteroides_finegoldii
s__Alistipes_finegoldii
s__Alistipes_sp_AP11
s__Peptostreptococcaceae_noname_unclassified
s__Bifidobacterium_bifidum
s__Prevotella_copri
s__Ruminococcus_lactaris
s__Bacteroides_salyersiae
s__Clostridium_innocuum
s__Bilophila_unclassified
s__Veillonella_parvula
s__Coprococcus_catus
s__Oxalobacter_formigenes
s__Alistipes_onderdonkii
s__Bacteroides_plebeius
−0.004−0.0020.000 0.002
EG values (impact on model output)
High
Feature value
Low
(b) HTOT EG Summary Plot (clinical features)
bmi
age
gender_female
race_afri
exercise
race_cauc
alcohol_drink
Cheese_num_week
Yogurt_num_week
sun_exposure
Probiotic_use
smoke
Milk_num_week
PC2
PC1
−0.10 −0.05 0.00 0.05 0.10 0.15
EG values (impact on model output)
High
Feature value
Low
HTOT EG Summary Plot (top 15 microbiome species)
s__Bifidobacterium_adolescentis
s__Bacteroides_cellulosilyticus
s__Ruminococcus_lactaris
s__Parabacteroides_goldsteinii
s__Catenibacterium_mitsuokai
s__Lachnospiraceae_bacterium_1_1_57FAA
s__Coprococcus_comes
s__Eubacterium_eligens
s__Bacteroides_stercoris
s__Peptostreptococcaceae_noname_unclassified
s__Parabacteroides_merdae
s__Coprobacillus_unclassified
s__Clostridiaceae_bacterium_JC118
s__Adlercreutzia_equolifaciens
s__Coprococcus_catus
−4 −2 0 2 4
EG values (impact on model output)
High
Feature value
Low
(c) STOT EG Summary Plot (clinical features)
bmi
age
race_afri
gender_female
Yogurt_num_week
exercise
alcohol_drink
Milk_num_week
sun_exposure
Cheese_num_week
smoke
Probiotic_use
race_cauc
PC1
PC2
−0.10 −0.05 0.00 0.05 0.10 0.15
EG values (impact on model output)
High
Feature value
Low
STOT EG Summary Plot (top 15 microbiome species)
s__Bacteroides_massiliensis
s__Odoribacter_splanchnicus
s__Eubacterium_siraeum
s__Adlercreutzia_equolifaciens
s__Alistipes_putredinis
s__Clostridium_symbiosum
s__Clostridium_clostridioforme
s__Bacteroides_caccae
s__Haemophilus_parainfluenzae
s__Bifidobacterium_adolescentis
s__Bacteroides_uniformis
s__Bifidobacterium_catenulatum
s__Parabacteroides_unclassified
s__Escherichia_unclassified
s__Clostridiales_bacterium_1_7_47FAA
−4 −2 0 2 4 6
EG values (impact on model output)
High
Feature value
Low

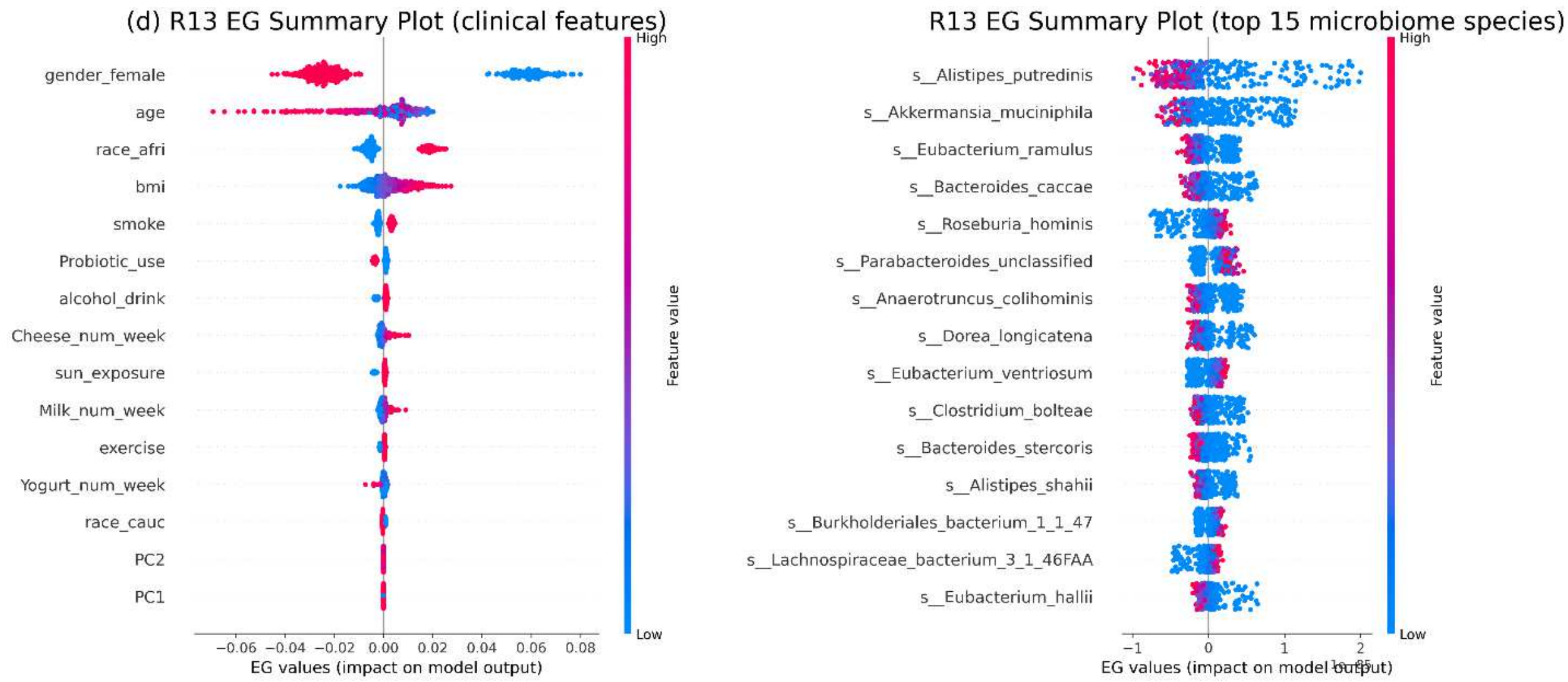


**Supplementary Figure S6. Gut microbiome species-level Expected Gradient (EG) attributions are individually small in magnitude compared to clinical variables across skeletal sites.** EG feature attributions are shown for the clinical variables and species-level microbiome input across (a) femoral neck (FNECK), (b) total hip (HTOT), (c) total spine (STOT), and (d) one-third radius (R13) BMD prediction tasks. The distributions illustrate that most individual species attributions are small in magnitude, despite measurable overall contribution from metagenomic relative abundance to predictive performance of the model. These results motivate downstream analysis of whether metagenomic predictive signals are organized into higher-order structure beyond isolated feature-level effects.

**(a) FNECK**

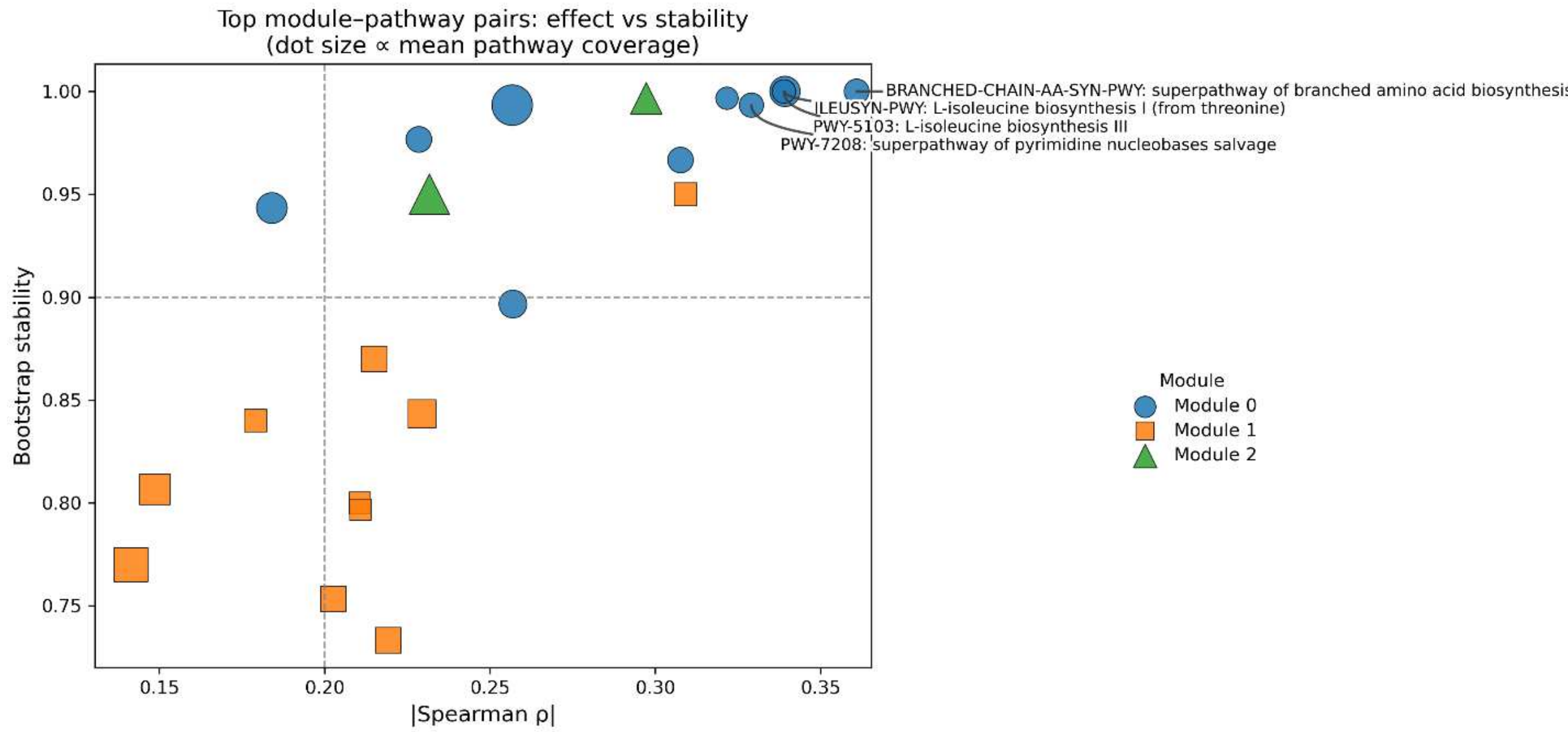


**(b) HTOT**

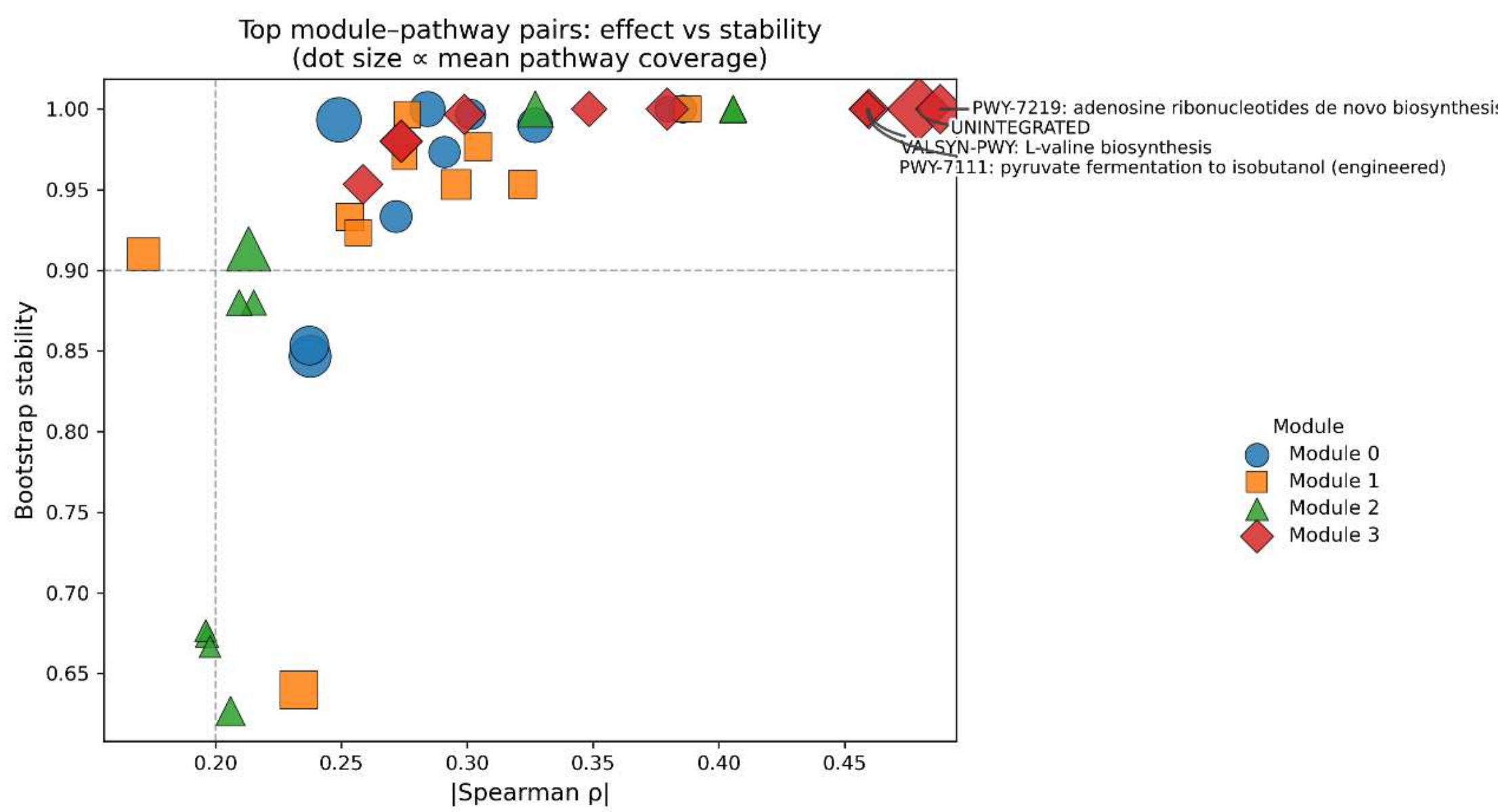


**(c) STOT**

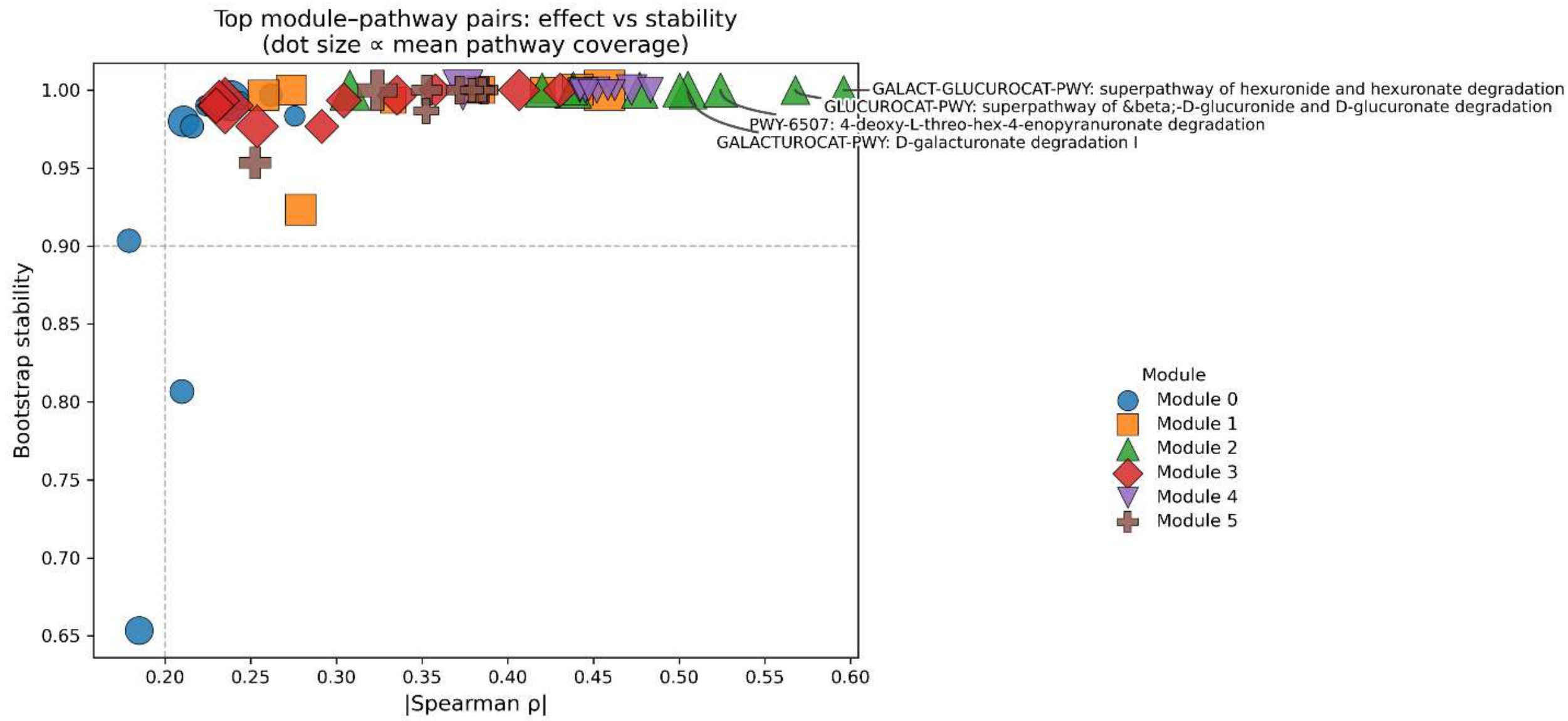


**(d) R13**

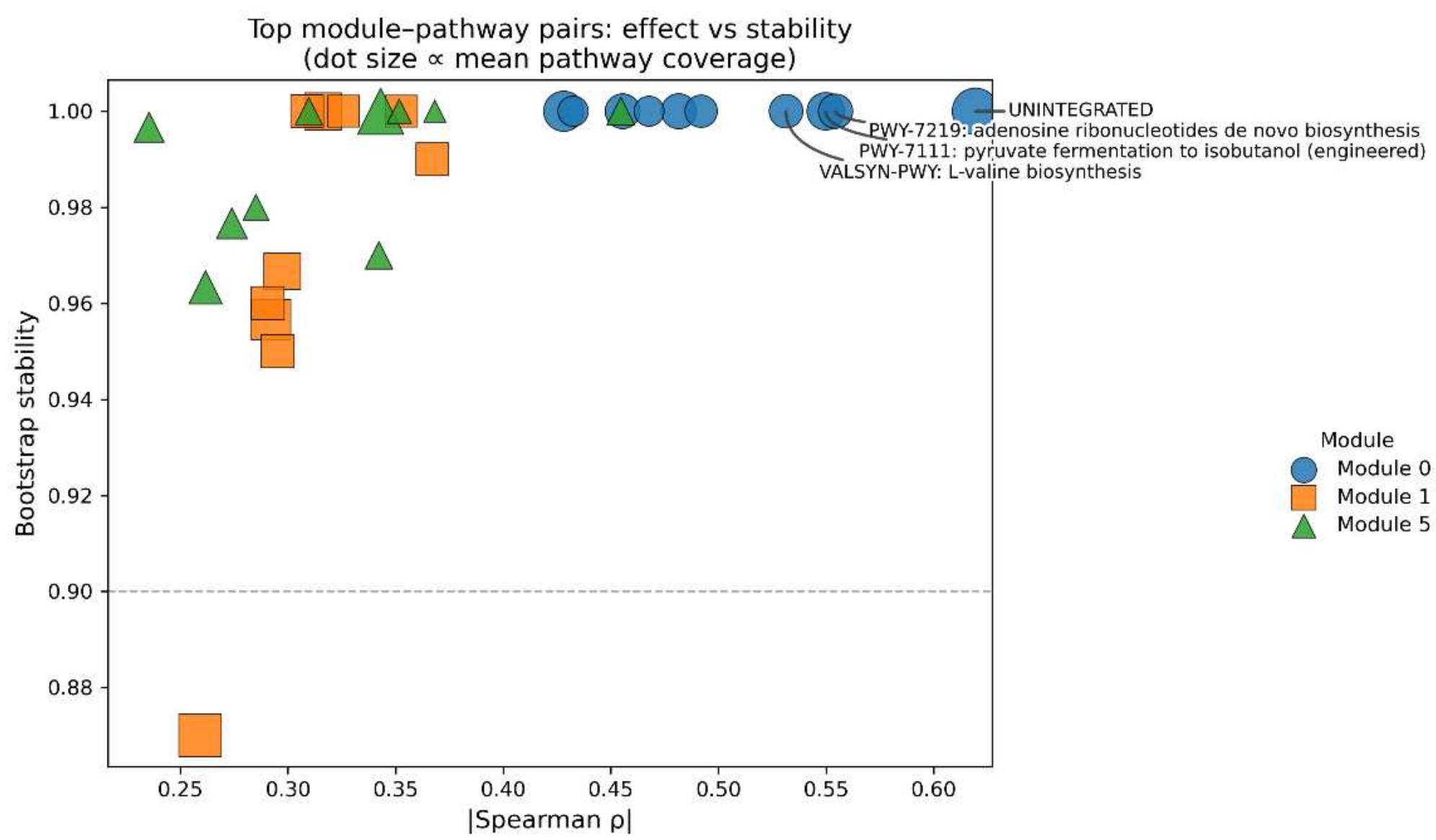


**Supplementary Figure S7. Effect–stability plots of top-ranked module–pathway associations across skeletal sites.** For **(a)** femoral neck (FNECK), **(b)** total hip (HTOT), **(c)** total spine (STOT), and **(d)** one-third radius (R13), the top 10 ranked module–pathway pairs for each module are shown using effect–stability plots derived from module-wise pathway association analysis based on

HUMAnN2-reconstructed pathway abundance and coverage profiles. The x axis shows the absolute Spearman correlation magnitude (|ρ|) between module-level Expected Gradient (EG) signals and pathway abundance across subjects, the y axis shows bootstrap stability, and point size indicates mean aggregated pathway coverage across subjects.
